# Working memory in technology-enhanced language learning: a systematic review from interactive to AI-mediated contexts

**DOI:** 10.3389/fpsyg.2026.1758104

**Published:** 2026-02-18

**Authors:** Xin Deng

**Affiliations:** Department of Public Foreign Language Teaching and Research, Jilin University of Finance and Economics, Changchun, China

**Keywords:** adaptive learning, AI-assisted language learning, aptitude–treatment interaction, cognitive load, cognitive load redistribution, computer-assisted language learning, multimodal instruction, working memory

## Abstract

**Introduction:**

Working memory (WM) is a central cognitive constraint in second and foreign language learning, particularly in technology-enhanced instructional environments. While pre-AI computer-assisted language learning (CALL) research has examined how interactive technologies interact with individual differences in WM capacity, the rapid emergence of AI-mediated language learning tools raises new questions about how WM demands are managed, redistributed, or compensated. This review examines how WM has been conceptualized and empirically addressed across two historical eras of language learning technology.

**Methods:**

This systematic review adopts a PRISMA 2020–compliant historical–comparative design and synthesizes 31 primary empirical studies, including 27 studies from the Interactive Era (2010–2024) and 4 studies from the AI-Mediated Era (2024–2025), supplemented by recent systematic reviews and theoretical work. Studies were analyzed within two analytically distinct corpora, focusing on instructional design features, WM-related outcomes, cognitive load management, and measurement approaches, followed by cross-era comparison guided by three research questions.

**Results:**

Interactive Era studies show that CALL, multimedia, and online platforms provide multimodal input, adaptive feedback, collaboration, and flexible pacing, but frequently induce cognitive overload and unequal learning outcomes associated with individual differences in WM capacity, which is typically treated as a fixed learner constraint. In contrast, AI-mediated studies reveal a qualitative shift. AI-assisted writing reduces lower-level encoding demands while increasing central-executive demands for evaluation and integration; biometric-adaptive reading systems preemptively regulate cognitive load and improve comprehension; and AI-orchestrated VR–AR vocabulary instruction yields large gains only within empirically bounded multimodal channel limits. AI-mediated data-driven learning further offloads corpus search, reallocating WM resources toward noticing and internalization.

**Discussion:**

Despite these advances, direct assessment of WM is largely absent from AI-mediated intervention studies, which rely on cognitive load proxies. This measurement gap limits causal inference regarding whether AI primarily reduces task demands, improves functional WM utilization, or supports WM capacity development. The review calls for future research to incorporate validated WM measures, adopt aptitude–treatment interaction designs, and establish evidence-based boundaries for AI-mediated multimodal adaptivity across diverse EFL and ESL contexts.

## Highlights

- Historical–comparative synthesis of 31 empirical studies spanning the Interactive Era (2010–2024; *n* = 27) and the emerging AI-Mediated Era (2024–2025; *n* = 4).
- Interactive technologies support WM through multimodality, adaptive feedback, and flexible pacing, yet frequently induce cognitive overload and produce unequal outcomes linked to individual WM capacity.
- AI-mediated tools redistribute—rather than merely reduce—cognitive load: generative AI offloads lower-level encoding while elevating central-executive demands for evaluation, prompt management, and integrative synthesis.
- Biometric-adaptive AI enables preemptive cognitive regulation; AI-orchestrated multimodal instruction shows optimal effects within an empirically bounded 3–4 channel limit.
- Critical measurement gap identified: direct WM assessment is virtually absent from AI intervention studies, precluding causal inference about whether AI reduces task demands, enhances WM utilization, or supports WM plasticity.

## 1 Introduction

The relationship between language learning technology and human cognition has long been framed around the limitations of working memory—the cognitive system responsible for the temporary storage and manipulation of information that underpins complex comprehension and production (Baddeley, 2000). In second language acquisition, working memory is widely recognized as a core bottleneck: tasks that overtax working memory tend to reduce accuracy, depth of processing, and long-term retention, particularly for learners with lower working memory capacity (Gathercole and Alloway, 2008). Consequently, the design of technology-enhanced language learning environments has historically been guided by the imperative to manage, or at least not exceed, this limited cognitive resource.

This review argues that the field is now in the midst of a transition between two technological eras with fundamentally different implications for working memory. The first is a pre-AI “Interactive Era,” in which technologies such as computer-assisted language learning software, hypermedia environments, captioned video, and online platforms provided valuable interactivity but were fundamentally static and pre-scripted (Lobin and Rösler, 2012). The second is an emerging “AI-Mediated Era,” in which generative AI chatbots, intelligent tutoring systems, biometric-adaptive platforms, and AI-orchestrated virtual and augmented reality environments are dynamic and dialogic, capable in principle of tailoring support to real-time learner states.

In the Interactive Era, interactivity was essentially reactive and bounded by pre-authored content, fixed branching, and rule-based feedback. Computer-assisted language learning software and online language courses grew in popularity, offering learners a range of interactive and engaging activities including multimedia presentations, language games, and virtual conversations (Jiang et al., 2017; Jones et al., 2017). However, these environments also presented challenges related to working memory: learners needed to manage cognitive load, cope with distractions, and navigate individual differences in working memory capacity to effectively learn new vocabulary, grammatical structures, and communication skills.

Within this paradigm, working memory was conceptualized primarily as a fixed individual-difference variable—a learner property that moderated the extent to which individuals could benefit from complex multimedia and self-directed tasks. Research in this era, especially under the influence of Cognitive Load Theory (Sweller et al., 2019), focused on mitigating the risk of overload by managing information density, controlling redundancy, sequencing tasks appropriately, and coordinating modalities to support dual-channel processing (Mutlu-Bayraktar et al., 2019). The underlying assumption was that good instructional design should stay within working memory limits; working memory itself was not something the system could sense, measure, or adapt to in real time.

Before proceeding, it is essential to distinguish among three theoretically and empirically distinct WM constructs that are often conflated in the literature (Unsworth and Engle, 2007; Gathercole and Alloway, 2008). WM capacity refers to the relatively stable, trait-like limit on the amount of information an individual can simultaneously maintain and manipulate, typically assessed through span tasks (e.g., digit span, reading span, operation span). WM utilization (or efficiency) refers to how effectively available WM resources are deployed under varying task conditions, including strategic allocation, attentional control, and resistance to interference—a process-level construct that can vary within individuals across contexts. WM training (or plasticity) refers to the potential for systematic practice to enhance WM capacity or efficiency, typically examined through extended training paradigms and transfer assessments. These three constructs carry distinct implications for instructional design: capacity sets an upper bound that instruction must respect, utilization determines how close to that bound learners can perform under given conditions, and plasticity determines whether instructional interventions can expand the bound itself. Critically, these distinctions have direct relevance for AI-mediated instruction, as AI systems might (a) respect capacity limits by dynamically adjusting task complexity, (b) improve utilization by optimizing scaffolding and reducing extraneous load, or (c) support plasticity through extended adaptive training regimens.

Empirical work in interactive environments repeatedly underscored this constraint. Varol and Erçetin (2021) showed that gloss type and position affected comprehension, with higher working memory capacity associated with better outcomes in hypermedia reading. Hong et al. (2021) reported that intrinsic cognitive load negatively affected flow experience, while gameplay interest positively affected flow in game-based environments. Türk and Erçetin (2014) found that interactive glosses were more effective than simultaneous glosses in promoting reading comprehension and incidental vocabulary learning, suggesting that better alignment with working memory limitations improves learning. Aryadoust (2020) reported that higher working memory capacity was associated with better performance on computerized while-listening tests. Across such studies, working memory consistently emerged as a predictor of success rather than a target for real-time intervention.

The arrival of powerful AI systems in language education is reshaping this landscape fundamentally. AI-mediated environments now include generative AI chatbots such as ChatGPT, Microsoft Copilot, and DeepSeek that can participate in open-ended dialogue, generate exemplars, and provide context-sensitive feedback. They also include adaptive learning platforms that adjust content difficulty, pacing, and support based on learner performance and, in advanced implementations, biometric indicators such as eye-tracking and physiological monitoring. Virtual and augmented reality environments combine immersive technologies with AI-driven content generation for multimodal, embodied language experiences (Squires, 2017). Intelligent personal assistants such as Amazon Alexa, Google Assistant, and Apple Siri enable voice-activated spoken interaction for listening and speaking practice.

These technologies are not limited to presenting pre-authored content; they can analyze learner input and behavior, detect patterns, and respond with adaptive scaffolding (Chen et al., 2020; Xu, 2025). This capability invites a reconceptualization of working memory from a static constraint to a dynamic target for adaptive compensation and regulation. Working memory becomes not only “something to design around” but potentially “something to design to”—a parameter that AI systems might measure, infer, and respond to. This evolution reintroduces the principle of Aptitude-Treatment Interaction, suggesting that AI systems could personalize instructional support based on a learner’s cognitive profile in ways previously impossible (Sana and Fenesi, 2025).

Initial empirical evidence points to both the promise and complexity of this technological shift. At a macro level, meta-analytic findings suggest that AI-assisted learning produces large overall effects (d = 1.17) relative to non-AI conditions, with particularly strong effects for vocabulary (d = 2.21) and receptive skills (d = 2.01). Notably, K–12 learners—whose working memory is still developing—show descriptively larger gains (d = 1.445) than college students (d = 0.988), a pattern consistent with a compensatory function of AI’s adaptive pacing for learners with less mature working memory capacity (Xu et al., 2025).

At a finer-grained level, recent AI-mediated interventions demonstrate that AI does not simply reduce cognitive load in the way traditional multimedia design sought to achieve; rather, it can redistribute load across working memory subsystems. Generative AI tools appear to offload lower-level encoding demands traditionally taxing the phonological loop while substantially increasing central executive demands for critical evaluation, prompt management, and integration of AI-generated content (Fan and Yao, 2025). Biometric-adaptive reading systems can preempt overload by adjusting difficulty in real time before errors accumulate (Yuan, 2025), while AI-driven multimodality enhances learning up to an empirically defined channel boundary of three to four concurrent sources, beyond which sensory overload undermines retention (Yu, 2025). AI-mediated data-driven learning can offload the search burden that traditionally taxed working memory in concordancing tasks, theoretically freeing resources for deeper noticing and internalization (Esfandiari and Allaf-Akbary, 2024).

Parallel evidence from neurocognitive research suggests that working memory itself may be more malleable than earlier static trait models implied. Computer-based multisensory working memory training using language materials has been shown to enhance both working memory capacity and second language ability, with transfer effects to novel auditory tasks and increased dorsolateral prefrontal cortex efficiency (Gkintoni et al., 2025). Additionally, evidence suggests that verbal, rather than spatial, working memory is particularly critical for second language achievement: high-achieving second language Chinese learners significantly outperformed low achievers on forward and backward digit span tasks but showed no differences in spatial working memory, with achievement correlating strongly with backward span (Xiao et al., 2025). These findings have direct implications for what aspects of working memory AI systems should prioritize scaffolding (Stakanova, 2023).

Despite these advances, a critical empirical gap tempers the promise of AI-mediated working memory support. Systematic reviews consistently document a measurement paradox: as AI in language education invokes working memory and cognitive load more explicitly, it measures working memory less directly. Chalmers et al. (2021), analyzing 111 s language aptitude studies spanning six decades, found working memory among the most frequently examined cognitive predictors (8.1% of independent variables) yet identified no AI-mediated interventions explicitly targeting or measuring working memory. Zhang and Aubrey (2024) found only one study examining working memory as an individual-difference factor across all second language pragmatics research, with no AI-mediated pragmatics studies including working memory measures. Goh and Aryadoust (2025) reported that while intelligent personal assistants and generative AI are increasingly used for second language listening and speaking, none of the reviewed AI studies included working memory tasks or aptitude-treatment interaction analyses. Xu (2025) noted that intelligent personal assistants are widely theorized to engage working memory through real-time communication practice, but direct empirical evidence on AI-mediated working memory outcomes remains absent.

Even the AI-mediated primary studies with the strongest cognitive focus rely on cognitive load scales and process indicators as proxies rather than direct working memory assessment. This creates a causal gap: without measuring working memory directly, researchers cannot determine whether AI reduces task demands (a design property), enhances functional working memory utilization (a processing property), or improves working memory capacity itself (a training property). Evidence that working memory may influence learning indirectly through proficiency (Teng, 2024) further complicates the picture, suggesting that working memory–AI relationships may involve mediation pathways that require sophisticated longitudinal and aptitude-treatment interaction designs to untangle.

Against this backdrop, the present systematic review is explicitly structured as a historical-comparative analysis of working memory in technology-enhanced language learning. To address the conceptual and empirical gaps identified above, the review is organized around three research questions:

RQ1 (Design Guidelines): What instructional design features of interactive language learning environments (2010–2024) support working memory efficiency, and which features challenge or overload working memory capacity?

RQ2 (WM × AI Affordances Interactions): How do AI-mediated language learning affordances—including generative chatbots, biometric-adaptive systems, and multimodal VR–AR platforms—interact with working memory processes, and do these interactions differ qualitatively from those observed in traditional interactive environments?

RQ3 (Boundary Conditions and Unintended Consequences): What are the empirical boundary conditions of AI-mediated working memory support, and what unintended consequences—including cognitive load redistribution, measurement gaps, and potential over-scaffolding (i.e., overprotection) with downstream implications for metacognitive monitoring and executive-skill development—emerge from AI integration in language learning?

Corresponding to these research questions, the review pursues three objectives. The first objective (aligned with RQ1) is to synthesize findings from a corpus of 27 empirical studies on the challenges and affordances of working memory in traditional, non-AI interactive language learning environments from 2010 to 2024, including computer-assisted language learning software, hypermedia, online platforms, and multimedia, thereby deriving evidence-based design guidelines for WM-sensitive instruction. The second objective (aligned with RQ2) is to juxtapose these findings with evidence from four recent AI-mediated primary empirical studies (Esfandiari and Allaf-Akbary, 2024; Fan and Yao, 2025; Yu, 2025; Yuan, 2025) that directly examine cognitive load and working memory-relevant processes in AI-assisted writing, adaptive reading, multimodal vocabulary learning, and AI-mediated data-driven learning, analyzing how AI affordances interact with WM subsystems in ways that differ from pre-AI technologies. The third objective (aligned with RQ3) is to identify the empirical boundary conditions under which AI-mediated support optimizes versus undermines WM functioning, and to document unintended consequences—particularly the cognitive load redistribution from encoding to evaluation, the measurement paradox whereby WM is increasingly invoked but decreasingly measured, and potential over-scaffolding (i.e., overprotection) with downstream implications for metacognitive monitoring and executive-skill development—that carry implications for future research and practice.

To systematize the relationship between WM constructs and AI affordances, Table 1 presents a conceptual framework mapping the three WM constructs (capacity, utilization, and training/plasticity) to four key AI affordances (adaptivity, multimodality, generative support, and feedback timing). For each pairing, the framework specifies the theorized mechanism and articulates a testable prediction suitable for future empirical investigation. For example, AI-driven adaptivity is hypothesized to respect capacity limits by dynamically adjusting task complexity, leading to the testable prediction that learners with lower WM capacity will show larger performance gains under adaptive versus fixed-difficulty conditions. Similarly, multimodal AI environments may improve WM utilization by distributing load across subsystems (phonological loop, visuospatial sketchpad), predicting that optimal retention will occur within a bounded channel range (e.g., 3–4 concurrent sources) with diminishing returns beyond this threshold. Generative AI support may offload lower-level encoding processes while increasing central-executive demands, predicting construct-specific load redistribution measurable via differentiated cognitive load instruments. Finally, preemptive feedback timing enabled by biometric AI may prevent utilization failures before they accumulate, predicting superior outcomes compared to reactive (post-error) adaptation. This framework enables researchers to formulate and test construct-specific hypotheses rather than treating WM as an undifferentiated variable, thereby advancing theoretical precision in the study of AI-mediated language learning.

**Table 1:** Corpus structure summary.

Corpus layer	Number of studies	Time period	Technology type	Working memory conceptualization
Interactive corpus	27	2010–2024	Traditional CALL, multimedia, online platforms, hypermedia, captioned video	Working memory as a constraint to design around; occasionally measured directly via span tasks
AI-mediated cluster	4	2024–2025	Generative AI chatbots, biometric-adaptive platforms, VR-AR with LLM integration, AI-mediated DDL	Working memory as a target for compensation and regulation; primarily assessed via cognitive load proxies and process data
Total primary empirical	31	2010–2025	–	–
Contextual literature	~10	2021–2025	Meta-analyses, systematic reviews, theoretical and neurocognitive papers	Interpretive context; documents the working memory measurement gap and motivates ATI-oriented research

CALL, computer-assisted language learning; VR-AR, virtual reality and augmented reality; LLM, large language model; DDL, data-driven learning; ATI, aptitude-treatment interaction.

The broader AI-related literature—including meta-analyses such as Xu et al. (2025), systematic reviews (Chalmers et al., 2021; Gkintoni et al., 2025; Goh and Aryadoust, 2025; Zhang and Aubrey, 2024), and theoretical frameworks (Sana and Fenesi, 2025; Xu, 2025)—is used to contextualize these 31 core empirical studies but is not counted in the primary empirical corpus. Additional empirical work examining working memory-relevant constructs in non-AI or AI-adjacent contexts, such as Teng (2024) and Xiao et al. (2025), further informs the interpretive framework.

By comparing the 27-study Interactive Era corpus with the 4-study AI-Mediated cluster and situating both within this broader context, the review seeks to identify continuities (what remains true across eras), transformations (what has fundamentally changed), and persistent measurement gaps (what researchers still cannot determine) in how working memory is conceptualized, engaged, and empirically assessed as language education transitions into the AI era.

## 2 Method

**Figure 1:**
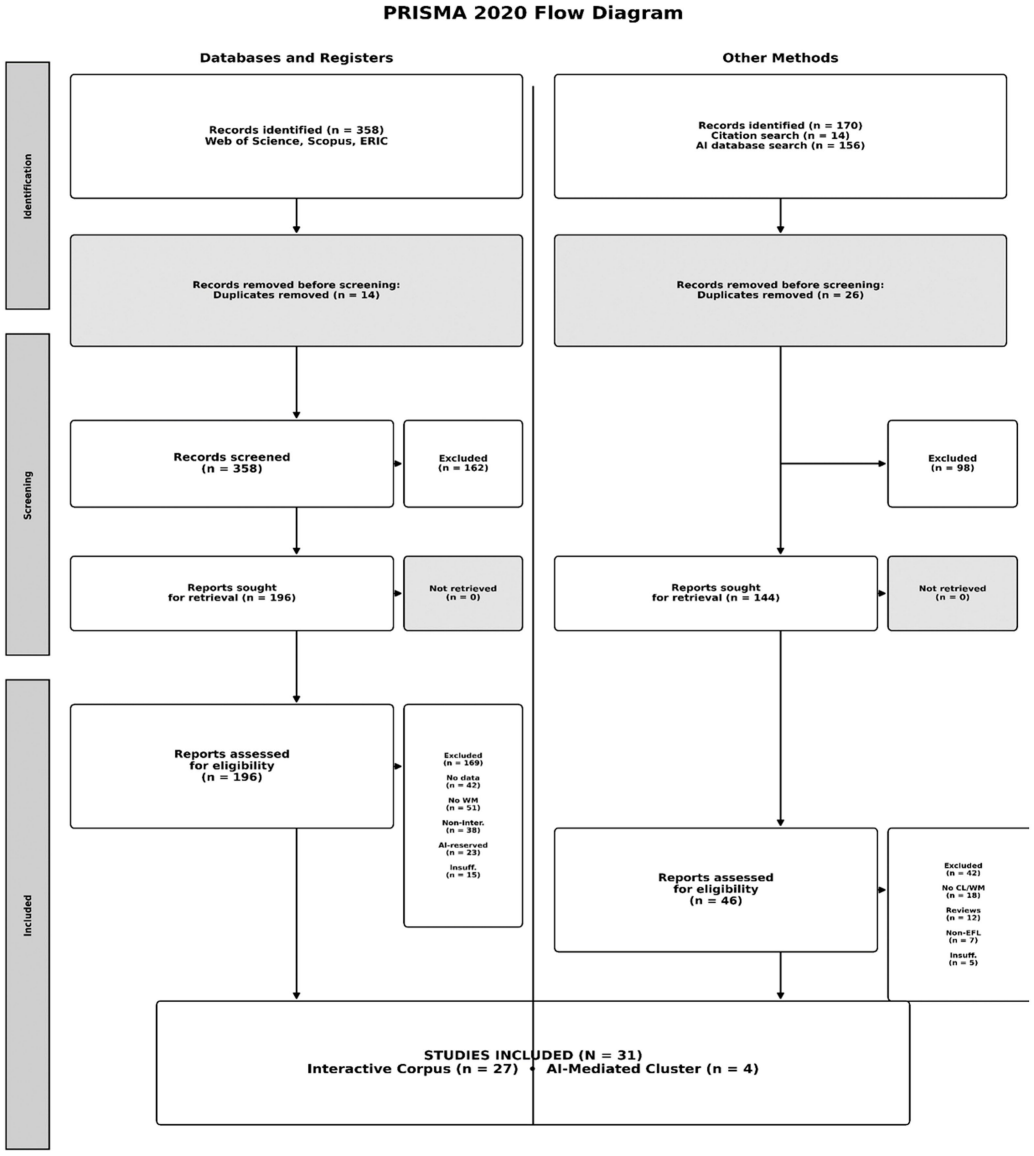
PRISMA 2020 flow diagram of study identification, screening, eligibility, and inclusion across phase 1 (interactive corpus) and phase 2 (AI-mediated cluster), including categorized reasons for full-text exclusions with exact counts.

This systematic review is reported in accordance with the Preferred Reporting Items for Systematic Reviews and Meta-Analyses (PRISMA) 2020 Statement (Page et al., 2021). Figure 1 provides the PRISMA 2020 flow diagram documenting identification, screening, eligibility, and inclusion across both Phase 1 (Interactive Corpus) and Phase 2 (AI-Mediated Cluster), including categorized reasons for full-text exclusion with exact counts. The completed PRISMA 2020 checklist with page/section cross-references is provided in Appendix A (PRISMA 2020 Checklist). Because the review is explicitly historical–comparative and constructs two analytically distinct corpora (Interactive vs. AI-mediated) rather than estimating a single pooled effect, synthesis is conducted within each corpus prior to cross-era comparison, and contextual (non-primary) literature used for interpretation is reported separately from the primary empirical study count.

This study adopts a two-phase systematic review design with an explicit historical-comparative structure. The goal is not to collapse all studies into a single pooled estimate but to construct two analytically distinct yet comparable corpora, each analyzed in relation to the three guiding research questions (RQ1: design guidelines; RQ2: WM × AI affordances interactions; RQ3: boundary conditions and unintended consequences): an Interactive Corpus of 27 empirical studies examining working memory in traditional, non-AI technology-enhanced language learning environments from 2010 to 2024, and an AI-Mediated Cluster of four empirical studies from 2024 to 2025 providing detailed evidence on cognitive load and working memory-relevant processes in AI-mediated English as a foreign language and second language learning. These 31 studies constitute the primary empirical dataset. Additional meta-analytic, systematic review, theoretical, and AI-adjacent empirical work is drawn upon to interpret patterns and highlight gaps but is not included in the numerical count of the primary empirical corpus.

Phase 1 focused on the pre-AI Interactive Era. A systematic search was conducted for studies published between 2010 and 2024 that investigated working memory in interactive, technology-enhanced language learning contexts. Searches were conducted in major databases including Web of Science, Scopus, and ERIC. The core search string combined working memory with generic interactive technology terms: “working memory” AND (“computer-assisted language learning” OR “interactive language learning” OR “online language learning”). This string was adapted to each database’s syntax while preserving its conceptual structure. Limits were set to peer-reviewed journal articles, English language, and second or foreign language learning contexts. Reference lists of eligible papers and key theoretical and review articles were screened through backward citation tracking to identify additional studies that met inclusion criteria but might not have been captured due to terminology variation.

In total, 372 records were initially identified through database searching and other sources (Figure 1). Titles and abstracts were screened for relevance, leading to the exclusion of 162 records that clearly did not concern working memory or technology-enhanced language learning. The remaining 196 full-text articles were assessed for eligibility against predefined inclusion and exclusion criteria. After this full-text screening, 169 articles were excluded (categorized reasons and counts are reported in Figure 1).

Studies were included in the Interactive Corpus if they reported empirical data (experimental, quasi-experimental, correlational, or mixed-methods) on language learning or performance in interactive, technology-enhanced environments such as computer-assisted language learning software, multimedia platforms, hypermedia, non-AI captioned video, or online courses. Studies were also required to explicitly discuss working memory, either conceptually by framing results in terms of working memory demands or capacity, or empirically by using working memory measures as predictors, moderators, or covariates in relation to language learning outcomes. Additionally, studies needed to provide sufficient methodological detail to allow assessment of sample characteristics, instructional context, technological environment, and outcome measures. Studies were excluded if they focused exclusively on non-interactive or purely traditional face-to-face instruction without technology, did not mention working memory or working memory-related constructs, were purely theoretical or descriptive without empirical data, or implemented AI-mediated instruction, as these were reserved for Phase 2 screening.

Applying these criteria yielded 27 empirical studies constituting the Interactive Corpus. These studies all predate the widespread deployment of generative AI and biometrically adaptive systems in language education and do not involve AI-mediated instruction. Technologies examined include conventional multimedia computer-assisted language learning, hypermedia environments, captioned video without AI, online learning platforms, language games, and traditional data-driven learning using concordancers.

Phase 2 targeted the emerging AI-Mediated Era, focusing on identifying primary empirical interventions that both implemented AI-mediated language learning and provided rich cognitive load or working memory-relevant data suitable for comparison with the Interactive Corpus. To capture the rapidly emerging AI landscape, the search strategy was expanded to include AI-specific terminology alongside working memory-related constructs. Searches were conducted through 2025 in the same core databases, using a representative string such as: (“working memory” OR “cognitive load”) AND (“artificial intelligence” OR “AI-assisted” OR “generative AI” OR “chatbot” OR “intelligent tutoring” OR “adaptive learning” OR “AI-mediated” OR “virtual reality language learning”). Additional targeted searches used combinations such as “cognitive load” AND “AI-assisted writing,” “biometric feedback” AND “language learning,” “VR” OR “AR” AND “AI” AND “vocabulary learning,” “LLM” AND “second language acquisition,” and “working memory training” AND “L2.” These searches were supplemented by forward and backward citation tracking from key AI-in-education and AI-in-second language acquisition publications, as well as manual scanning of recent issues of high-relevance journals including Computer Assisted Language Learning, Language Learning & Technology, ReCALL, and Frontiers in Psychology. Identification, screening, eligibility, and inclusion counts for Phase 2 are reported in Figure 1 to document the full two-phase selection process in PRISMA 2020 format.

To retain a tight, analytically coherent cluster for detailed comparative analysis, Phase 2 applied more restrictive criteria. Studies were included in the AI-Mediated Cluster if they implemented AI-mediated language learning interventions such as generative AI-assisted writing, AI-orchestrated virtual and augmented reality instruction, biometric-adaptive AI reading platforms, or AI-mediated data-driven learning. Studies were also required to report quantitative data on cognitive load and/or working memory-relevant processing, including validated cognitive load scales, detailed subscale analyses, or process indicators such as eye-tracking interpreted in terms of working memory demands. Additionally, studies needed to involve English as a foreign language or second language learners in classroom-like or ecologically valid learning settings, and to provide sufficient statistical detail including means, standard deviations, effect sizes, F-statistics, partial eta squared values, and beta coefficients to support comparative analysis. Studies were excluded if they were systematic reviews, meta-analyses, or theoretical papers without primary empirical data, as these were used for contextual interpretation only. Studies were also excluded if they did not include cognitive load or working memory-relevant measures or examined AI in non-language-learning contexts. Based on these criteria, four primary empirical studies were retained as the AI-Mediated Cluster (Figure 1).

Yu (2025) conducted a pretest-posttest randomized controlled trial with 383 Chinese English as a foreign language learners examining AI-driven multimodal vocabulary instruction using virtual and augmented reality technologies including Unity, Oculus Quest 2, and Google MediaPipe combined with a generative AI chatbot based on ChatGPT-4. The study provides quantitative data on sensory overload boundaries and establishes an inverted-U relationship between channel complexity and retention. Yuan (2025) conducted a 12-week randomized controlled trial with 300 Chinese English as a foreign language learners investigating an AI-enhanced biometric-adaptive reading platform called Smart Sparrow that integrates eye-tracking using Tobii Pro X3-120 and physiological indicators to dynamically adjust text difficulty. The study demonstrates preemptive cognitive regulation and provides detailed cognitive load data from the Differentiated Cognitive Load Questionnaire subscales alongside eye-tracking indicators. Fan and Yao (2025) conducted a scale development and validation study with 546 participants across exploratory and confirmatory factor analysis samples for the Cognitive Load Scale for AI-Assisted L2 Writing, examining Chinese English as a foreign language learners using a generative AI chatbot called DeepSeek V3.1 for argumentative writing. The study provides a detailed four-factor profile of cognitive load redistribution across Authorial Core Processing, Critical Evaluation, Prompt Management, and Integrative Synthesis. Esfandiari and Allaf-Akbary (2024) conducted a three-arm randomized controlled trial with 93 Iranian advanced English as a foreign language learners comparing AI-mediated intelligent data-driven learning using Microsoft Copilot based on GPT-4, traditional corpus-based data-driven learning using AntConc, and conventional instruction for developing interactional metadiscourse markers. The study demonstrates very large effects for AI-mediated data-driven learning and theorizes working memory support via Schmidt’s noticing hypothesis.

To provide interpretive context for the 31-study empirical corpus, additional sources were systematically reviewed but not included in the numerical study count. These include meta-analytic work such as Xu et al. (2025), who synthesized 15 AI-assisted second language learning studies with 2,156 participants and reported large overall effects and potential developmental differences consistent with working memory compensation. Systematic and narrative reviews documenting the working memory measurement gap and AI’s cognitive implications were also consulted, including Chalmers et al. (2021), Zhang and Aubrey (2024), Goh and Aryadoust (2025), and Gkintoni et al. (2025). Theoretical and conceptual papers elaborating aptitude-treatment interaction frameworks and AI-related aptitude perspectives were reviewed, including Sana and Fenesi (2025) and Xu (2025). Empirical AI-adjacent studies with working memory-relevant data that inform but are not central to the two main corpora were also considered, including Teng (2024) and Xiao et al. (2025). These sources inform the Discussion and Future Directions sections, particularly the articulation of the measurement paradox and the argument for aptitude-treatment interaction-based AI research, but they do not alter the primary empirical synthesis count of 31 studies.

For each of the 31 empirical studies comprising 27 Interactive and 4 AI-Mediated, a standardized data-extraction template was used to capture bibliographic information including authors, year, journal, and country or region. Participant characteristics were recorded including sample size, age and educational level, first language, target language, and proficiency indicators. Instructional context was documented including course type, skills targeted, and duration and intensity of the intervention. Technology characteristics were captured including type of platform or tool, whether non-AI or AI-mediated, and presence of multimodality, adaptivity, or biometric integration. Working memory-related constructs and measures were recorded including direct working memory tests such as span tasks and n-back, cognitive load scales such as NASA-TLX, the Differentiated Cognitive Load Questionnaire, and the Cognitive Load Scale for AI-Assisted L2 Writing, and process indicators such as eye-tracking and physiological measures interpreted in terms of working memory demands. Study design was documented including experimental, quasi-experimental, correlational, scale development and validation, or mixed methods. Outcome measures were recorded including language performance such as vocabulary, reading comprehension, and writing quality, process indicators, and affective outcomes. Key findings were extracted specifically those related to working memory challenges such as overload and inequitable effects by working memory level and working memory affordances such as strategic use of working memory, dynamic regulation, and compensation.

Analysis proceeded in two stages. In the within-era synthesis stage, for the Interactive Corpus, themes were identified concerning how working memory was conceptualized as constraint, predictor, or moderator, how it was measured directly versus inferred, and how interactive design features affected working memory-related outcomes in terms of affordances versus overload. For the AI-Mediated Cluster, the focus was on how AI systems redistributed cognitive load across working memory subsystems, how they regulated working memory demands through reactive versus preemptive mechanisms, and what specific boundary conditions emerged such as channel limits in multimodal virtual and augmented reality instruction.

In the historical-comparative integration stage, findings from the two corpora were compared along dimensions defined by the conceptual framework, including working memory as constraint versus working memory as target for compensation and regulation, load reduction versus load redistribution, reactive adaptation versus preemptive regulation via biometrics, direct working memory measurement versus reliance on cognitive load proxies and process indicators, and general notions of overload risk versus empirically specified boundary conditions such as three to four concurrent channels in virtual and augmented reality instruction. This comparative structure underpins the Results, Discussion, and Implications sections, where the evolution of English as a foreign language education from static interactive environments to AI-mediated systems is analyzed in terms of continuities, transformations, and persistent empirical gaps regarding working memory.

The quality of the selected studies was assessed using the Mixed Methods Appraisal Tool (Pluye et al., 2011), which is designed to evaluate qualitative, quantitative, and mixed-methods research. The Mixed Methods Appraisal Tool was used to appraise study design, data collection, analysis, and reporting. For randomized controlled trials within both corpora, additional evaluation employed the Cochrane Risk of Bias tool (Higgins et al., 2021). For non-randomized quantitative studies, the Risk of Bias in Non-randomized Studies of Interventions tool (Sterne et al., 2016) guided assessment of confounding, selection, measurement, and reporting biases.

Notable quality considerations, especially pertinent to the AI-Mediated Cluster and contextual meta-analytic work, include evidence of publication bias favoring positive AI effects, as indicated by funnel plot asymmetry in the meta-analysis by Xu et al. (2025). The concentration of AI-mediated primary studies in EFL contexts, specifically Chinese and Iranian learners, limits generalizability to other languages, regions, and educational systems. The persistent absence of direct working memory measurement in AI-mediated intervention studies, despite heavy reliance on working memory-related theorizing, represents a significant methodological limitation. Quality assessment was conducted independently by two reviewers, with discrepancies resolved through discussion and consensus (Table 2).

**Table 2:** Corpus structure summary.

Corpus layer	Number of studies	Time period	Technology type	Working memory conceptualization
Interactive corpus	27	2010–2024	Traditional CALL, multimedia, online platforms, hypermedia, captioned video	Working memory as a constraint to design around; occasionally measured directly via span tasks
AI-mediated cluster	4	2024–2025	Generative AI chatbots, biometric-adaptive platforms, VR-AR with LLM integration, AI-mediated DDL	Working memory as a target for compensation and regulation; primarily assessed via cognitive load proxies and process data
Total primary empirical	31	2010–2025	—	—
Contextual literature	~10	2021–2025	Meta-analyses, systematic reviews, theoretical and neurocognitive papers	Interpretive context; documents the working memory measurement gap and motivates ATI-oriented research

CALL, computer-assisted language learning; VR-AR, virtual reality and augmented reality; LLM, large language model; DDL, data-driven learning; ATI, aptitude-treatment interaction.

These methodological foundations support the subsequent comparative analysis of how working memory is challenged, supported, and in AI contexts dynamically reconfigured across the two technological eras.

## 3 Results

The review synthesized 31 primary empirical studies examining the challenges and affordances of working memory (WM) in technology-enhanced language learning. Twenty-seven studies formed the Interactive Corpus, representing traditional computer-assisted language learning (CALL) software, multimedia platforms, hypermedia environments, captioned video, and online learning systems published between 2010 and 2024. Four studies formed the AI-Mediated Cluster, representing generative AI chatbots, biometric-adaptive reading platforms, and VR–AR systems with large language model (LLM) integration published between 2024 and 2025. Across the corpus, research designs included randomized controlled trials, quasi-experimental studies, correlational investigations, scale development and validation work, and mixed-methods designs, conducted in classrooms, online platforms, language laboratories, and immersive virtual environments with learners of varied ages, proficiency levels, and first-language backgrounds.

The results are organized to foreground both the established patterns from traditional interactive environments and the emerging patterns from AI-mediated contexts. For each theme, findings from the 27 Interactive Corpus studies are presented first, followed by examination of how these patterns are transformed, extended, or problematized in the AI-Mediated Cluster and related recent reviews. Table 3 provides a study-level comparative evidence map of all included primary empirical studies (*n* = 31) and serves as the traceable basis for the cross-era synthesis reported in the Results section.

**Table 3:** Comparative analysis of the 31 included studies (*n* = 31).

Study (Author, year)	Technology type	Era	WM conceptualization	WM measurement approach	Outcome measures	Key findings
Aryadoust (2020)	Computerized while-listening test + eye-tracking	Interactive	WM capacity as predictor of computerized listening performance	Indirect: eye-tracking/behavioral indicators; WM invoked as individual difference	Listening performance; gaze metrics; answer changes	Higher WM capacity aligned with more efficient processing and stronger performance.
Baralt (2015)	Online language teaching/CMC with recasts	Interactive	WM capacity as moderator of feedback uptake under complexity	Direct: WM capacity measure (span/complex WM)	L2 development after recasts; interactional performance	WM capacity and cognitive complexity jointly shaped responsiveness to recasts.
Cevik and Altun (2016)	Computer-assisted complex task with instructional strategy manipulation	Interactive	WM as resource underpinning complex task performance; strategy–WM fit	Direct: WM performance measure(s)	Task performance (accuracy/efficiency)	Task outcomes varied by WM performance and instructional strategy.
Denhovska et al. (2016)	Incidental L2 grammar learning under controlled input conditions	Interactive	WM as individual-difference constraint on incidental acquisition	Direct: WM measure used as predictor/moderator	Grammar learning indices	Higher WM supported incidental grammar acquisition under frequency exposure.
Hong et al. (2021)	Game-based environment (remote association game)	Interactive	Cognitive load as WM-demand factor shaping engagement (flow)	Indirect: intrinsic cognitive load self-report	Flow; performance progress during gameplay	Higher intrinsic load reduced flow; gameplay interest increased flow.
Huffman and Hahn (2017)	Memory-enhancement procedures in foreign language learning tasks	Interactive	WM bottlenecks addressed via encoding/memory supports	Not direct WM: WM discussed conceptually	Vocabulary/learning performance; retention	Optimized memory procedures improved learning outcomes consistent with reduced WM bottlenecks.
Hwang et al. (2012)	Personalized educational computer game (adaptive to learning styles)	Interactive	Design intended to manage cognitive load within WM limits	Indirect/None: no direct WM task reported	Learning achievement; game-based performance	Personalized game design improved outcomes consistent with better cognitive fit.
Hwang et al. (2013)	Inquiry-based mobile learning model	Interactive	Cognitive load as proxy for WM demand in mobile inquiry	Indirect: cognitive load scale	Learning achievement; cognitive load	Inquiry-based mobile model influenced achievement and cognitive load profiles.
Jiang et al. (2017)	ELT multimedia courseware (multimedia learning environment)	Interactive	Multimedia design as determinant of WM load (modality/segmentation)	Indirect: learner-evaluated multimedia design/cognitive load indicators	Courseware evaluation; learning/performance indices	CTML-aligned courseware features were associated with better learning usability and reduced overload.
Jones et al. (2017)	Smartphone-supported migrant language learning (field trial)	Interactive	Mobile micro-learning to reduce WM burden and support sustained engagement	Not direct WM: WM discussed conceptually	Engagement/use patterns; language learning outcomes (field)	Smartphone design supported participation; task structure mitigated cognitive burden *in situ*.
Kozan et al. (2015)	Multimedia L2 text comprehension (input modality manipulation)	Interactive	WM capacity as moderator of modality effects in multimedia reading	Direct: WM capacity measure (span-based)	L2 text comprehension	Input modality effects on comprehension depended on WM capacity; optimal modality reduced overload.
Kukulska-Hulme (2019)	Mobile language learning in migrant contexts	Interactive	Mobile design to manage attentional/Wm demands in real-world learning	Not direct WM: conceptual framing	Design/implementation implications for mobile L2 learning	Highlights mobile design principles that reduce cognitive burden in authentic contexts.
Lee (2021)	Multimodal multiple-document reading + automated reflection report	Interactive	Scaffolding to support WM during multimodal integration	Indirect: performance + process prompts; WM inferred	Reading/integration performance; epistemic cognition indicators	Epistemic prompting and automated reflection supported multimodal integration and performance.
Lin et al. (2022)	Augmented-reality ubiquitous writing application (EFL)	Interactive	AR scaffolding to support WM during writing processes	Indirect: performance indicators; WM invoked via cognitive load rationale	Writing quality/performance	AR-supported writing improved outcomes consistent with scaffolded processing.
Makransky et al. (2016)	Virtual simulation environment (immersive learning)	Interactive	Immersive simulation as source of cognitive load with potential skill gains	Indirect/None: WM inferred from learning performance	Skill learning and performance outcomes	Simulation-based preparation improved skill performance while highlighting cognitive demands of immersion.
Scharinger et al. (2023)	Gamified n-back WM task (EEG + eye-tracking)	Interactive	WM training/performance under gamification; attentional control	Direct: n-back performance; physiological/eye-tracking indices	n-back accuracy/RT; EEG; eye-tracking	Gamification altered engagement and neurocognitive markers with mixed implications for WM training efficiency.
Schumacher and Ifenthaler (2018)	Learning analytics-informed design (higher education)	Interactive	Motivational dispositions as design inputs to optimize cognitive processing	Not direct WM: conceptual/empirical analytics framing	Learner dispositions; analytics-informed design implications	Motivational profiles inform analytics-driven personalization to support effective cognitive allocation.
Silva Barbosa et al. (2023)	Gamified logic tutoring system (stereotyped gamification)	Interactive	Design features as determinants of cognitive-affective load	Indirect: affective/behavioral outcomes	Negative thinking; learning/performance in tutoring	Stereotyped gamification increased negative thinking, implying added non-instructional load.
Teng (2019)	Captioned videos for ESL comprehension	Interactive	WM capacity as moderator of caption benefits	Direct: WM measure (span task)	Video comprehension	Captions supported comprehension with stronger benefits under adequate WM resources.
Teng (2023)	Captioned video for incidental vocabulary learning	Interactive	WM capacity as predictor/moderator of incidental vocabulary gains	Direct: WM measure (span task)	Incidental vocabulary learning/retention	WM contributed to vocabulary learning and retention from captioned input.
Teng (2024)	Captioned video genres (incidental vocabulary)	Interactive	WM influences learning directly and indirectly via proficiency	Direct: Reading Span Task; proficiency as mediator	Vocabulary gains; proficiency	WM predicted proficiency, which in turn predicted vocabulary gains across video genres.
Türk and Erçetin (2014)	Multimedia glosses (interactive vs. simultaneous) in L2 reading	Interactive	Presentation format to align demands with WM limits	Indirect/Direct: WM capacity considered in interpretation; gloss design reduces split attention	Reading comprehension; incidental vocabulary learning	Interactive gloss display improved comprehension and vocabulary relative to simultaneous display.
Varol and Erçetin (2021)	Electronic reading with gloss type/position manipulation	Interactive	WM capacity moderates effectiveness of gloss designs	Direct: WM capacity measure	L2 reading comprehension	Gloss type and position interacted with WM capacity to influence comprehension.
Weissheimer et al. (2019)	Gamified L2 vocabulary learning (Vocabox)	Interactive	Engagement and cognitive demands in gamified vocabulary learning	Not direct WM: WM invoked conceptually	Vocabulary learning outcomes	Gamification supported vocabulary learning; design choices implicated cognitive load management.
Xu and Xia (2021)	Computer keystroke logging + process graphs in L2 writing	Interactive	Process scaffolding to reduce WM burden in writing development	Indirect: keystroke/process indicators	Writing process metrics; writing development outcomes	Process scaffolding supported writing development and clarified WM-intensive subprocesses.
Yang et al. (2019)	ANN-based computational modeling of cognitive abilities for English acquisition	Interactive	WM-related cognitive abilities as predictors in individualized modeling	Indirect: modeled cognitive abilities (including WM-related inputs)	Predicted acquisition/performance indices	Modeling captured individual cognitive profiles relevant to acquisition, supporting personalization logic.
Zhonggen et al. (2019)	Mobile learning platform	Interactive	Cognitive load as proxy for WM demand in mobile learning	Indirect: cognitive load scale	Satisfaction; learning outcomes; cognitive load	Platform design influenced satisfaction and learning with measurable cognitive load differences.
Esfandiari and Allaf-Akbary (2024)	AI-mediated intelligent data-driven learning (Microsoft Copilot) vs. AntConc vs. control	AI-mediated	AI offloads search burden; frees WM for noticing/internalization	Indirect: performance outcomes; WM inferred (no direct WM task)	EFL writing (interactional metadiscourse markers)	AI-mediated DDL produced very large writing gains relative to traditional DDL and control.
Fan and Yao (2025)	Generative AI-assisted L2 writing (DeepSeek) + scale development	AI-mediated	Load redistribution from encoding to evaluation/management (central executive)	Indirect: validated cognitive load scale (CL-AI-L2W)	Cognitive load subscales; writing-related process demands	Critical evaluation, prompt management, and synthesis loads exceeded encoding load; robust factor structure.
Yu (2025)	AI-driven VR-AR multimodal vocabulary instruction (ChatGPT-4 integration)	AI-mediated	AI enhances learning within channel-capacity limits; overload boundary conditions	Indirect: cognitive load/overload surveys; process-performance linkage	Vocabulary posttest/delayed posttest; overload indices	Large vocabulary gains with inverted-U channel effect; overload increased beyond 3–4 channels.
Yuan (2025)	Biometric-adaptive AI reading platform (Smart Sparrow + eye-tracking)	AI-mediated	Preemptive regulation of WM demands via biometric-based adaptation	Indirect: Differentiated Cognitive Load Questionnaire + eye-tracking indicators	Reading comprehension; cognitive load; eye-tracking metrics	Adaptive biometric regulation reduced reported load and improved comprehension relative to non-adaptive control.

This evidence-mapping table codes each primary empirical study by technology type, era (Interactive vs. AI-mediated), working-memory (WM) conceptualization, WM measurement approach (Direct = psychometric WM task; Indirect = cognitive-load (CL) scale/process proxy; None = WM invoked without an assessment), outcome measures, and WM-relevant findings. AI, artificial intelligence; ANN, artificial neural network; AR, augmented reality; CALL, computer-assisted language learning; CL, cognitive load; CL-AI-L2W, Cognitive Load Scale for AI-Assisted L2 Writing; CMC, computer-mediated communication; CTML, cognitive theory of multimedia learning; DDL, data-driven learning; EEG, electroencephalography; EFL, English as a foreign language; ESL, English as a second language; L2, second language; VR, virtual reality; VR-AR, virtual/augmented reality; WM, working memory.

This table evidence-maps all included primary empirical studies using consistent coding rules to support transparent cross-era comparison. “WM measurement approach” is coded as Direct (psychometric WM task), Indirect (proxy indicators such as cognitive load scales and/or process measures interpreted as WM demand), or None (WM invoked conceptually without an empirical WM/CL operationalization). WM = working memory; AI = artificial intelligence.

### 3.1 Challenges

Across the corpus, WM-related challenges manifested in both traditional and AI-mediated environments, but with distinct profiles in each technological era.

In the Interactive Corpus, the most frequently documented challenge was cognitive load and information processing. Interactive environments routinely presented multiple streams of information—text, audio, images, animations, hyperlinks—often simultaneously (Lusk et al., 2009). When sequencing was suboptimal or scaffolding insufficient, these conditions exceeded learners’ limited WM capacity, resulting in split attention, disorientation, and reduced learning outcomes (Kozan et al., 2015). Learners with lower WM capacity were particularly disadvantaged under dense multimedia conditions, confirming core predictions of Cognitive Load Theory that learning deteriorates when intrinsic and extraneous load together exceed available resources.

Distractions and interruptions constituted a second pervasive challenge. Online and multimedia platforms made task-switching effortless, inviting off-task browsing, rapid window-shifting, and multitasking (Jones et al., 2017; Kukulska-Hulme, 2019). From an attentional control perspective, these environments diluted the sustained focus required for WM-dependent processing: learners who frequently shifted attention exhibited weaker retention and less accurate performance on comprehension and production tasks.

A third pattern concerned individual differences in WM capacity. Studies repeatedly showed that learners varied markedly in WM due to age, cognitive abilities, and prior language experience (Denhovska et al., 2016). In line with the working memory model and related empirical work (Cevik and Altun, 2016; Unsworth and Engle, 2007; Varol and Erçetin, 2021; Weissheimer et al., 2019), individuals with higher WM capacity were more likely to benefit from complex hypermedia tasks, interactive glosses, and self-paced online activities, while lower-WM learners showed steeper performance decrements as task complexity and information density increased.

Language proficiency and task complexity interacted systematically with WM. When linguistic materials involved dense vocabulary, complex morphosyntax, or rapid input, lower-proficiency learners’ WM capacity was quickly saturated, particularly in multimedia and hyperlinked environments (Makransky et al., 2016). Intrinsic cognitive load rose with linguistic complexity; in the absence of graded scaffolding, this produced floor effects for lower-proficiency learners and widened performance gaps between higher- and lower-WM profiles.

The corpus also documented technical difficulties and glitches as nontrivial WM stressors. Slow connections, system crashes, and unintuitive interfaces introduced extraneous cognitive load, diverting WM resources away from linguistic processing and toward troubleshooting (Golonka et al., 2014; Hwang et al., 2013; Sweller et al., 2019). Learners repeatedly reported frustration and demotivation when technical issues co-occurred with demanding tasks.

Finally, anxiety and stress were shown to modulate WM functioning in technology-rich tasks. High-stakes computer-based tests, unfamiliar platforms, and visible performance metrics sometimes increased anxiety, which in turn impaired WM and task performance (Dörnyei and Ushioda, 2021). Consistent with the Yerkes–Dodson law, moderate arousal occasionally enhanced engagement, but sustained high stress undermined WM-dependent comprehension, production, and problem solving (Ionescu and Vasc, 2014).

The AI-Mediated Cluster preserves many of these challenges but adds new layers of complexity. One important continuity—with added precision—is cognitive overload in multimodal environments. Whereas traditional studies treated overload as a diffuse risk in information-rich contexts, AI-mediated research begins to specify its quantitative boundary conditions. In a randomized controlled trial with 383 Chinese EFL learners, Yu (2025) compared AI-driven VR–AR multimodal vocabulary instruction (Unity, Oculus Quest 2, Google MediaPipe plus ChatGPT-4) to non-AI conditions. The AI multimodal group obtained much higher vocabulary scores than the control group (posttest M = 137.00 vs. 76.05; Hedges’ g = 1.24) and maintained substantial gains at delayed posttest (M = 129.00). However, 61% of learners in the AI multimodal condition reported sensory overload, and overload scores were inversely correlated with retention (*β* = −0.53, *p* = 0.003). An inverted-U relationship emerged, with optimal performance at approximately three to four concurrent information channels (R^2^ = 0.41), a pattern consistent with capacity estimates in short-term memory research (Cowan, 2001); beyond this threshold, WM appeared overwhelmed despite AI’s adaptive capabilities. This pattern empirically confirms channel limits that earlier CALL research had mainly theorized.

More fundamentally, AI-mediated environments alter not only the amount but also the distribution of cognitive load. Whereas traditional designs sought to reduce total load by optimizing sequencing and modality, AI systems often redistribute load across WM subsystems. Fan and Yao (2025) developed and validated the Cognitive Load Scale for AI-Assisted L2 Writing (CL-AI-L2W; *α* = 0.94) with 546 Chinese university EFL learners using the generative AI chatbot DeepSeek V3.1 for argumentative writing. Their four-factor model showed that Authorial Core Processing—lexical retrieval and grammatical encoding—had the lowest mean load (M = 3.48 on a 7-point scale), whereas Critical Evaluation of AI-generated content had the highest (M = 4.81), followed by Prompt Management (M = 4.55) and Integrative Synthesis (M = 4.40), with excellent fit indices (CFI = 0.97, RMSEA = 0.059). In contrast to the Interactive Corpus, where WM was primarily taxed at the level of input processing (phonological loop and visuospatial sketchpad), AI-assisted writing tasks shifted the main burden to central-executive functions—monitoring, verification, and integrative reasoning. Learners lacking metacognitive or evaluative strategies may thus experience AI support as cognitively more demanding, even when encoding load is reduced (Li, 2023).

A cross-cutting challenge that became particularly salient in the AI-Mediated Cluster is the empirical measurement gap. Despite six decades of L2 aptitude research establishing WM as a key predictor, AI studies rarely measure WM directly. Chalmers et al. (2021) reported that WM was among the most frequently examined cognitive predictors (8.1% of independent variables) in 111 aptitude studies, yet they identified no AI-mediated interventions explicitly targeting or measuring WM. Zhang and Aubrey (2024) found only one study including WM as an individual-difference factor across the entire L2 pragmatics literature, with no AI-mediated pragmatics studies assessing WM. Goh and Aryadoust (2025) showed that research on intelligent personal assistants and generative AI for L2 listening and speaking almost never incorporates WM tasks or aptitude–treatment interaction analyses. Xu (2025) similarly noted that intelligent personal assistants are widely theorized to engage WM through real-time conversational practice, but direct empirical evidence of AI-mediated WM outcomes is lacking. Even in the AI-mediated primary studies with the strongest cognitive focus—Yuan (2025), Fan and Yao (2025), and Yu (2025)—WM remains an inferred construct; cognitive load scales (Differentiated Cognitive Load Questionnaire, CL-AI-L2W, sensory overload surveys) and process indicators (eye-tracking) are used as proxies for WM engagement. By contrast, several Interactive Corpus studies employed direct WM assessments (e.g., digit spans, reading span tasks). As a result, just as AI research invokes WM and cognitive load more explicitly, it paradoxically measures WM less directly, creating a causal gap: it remains unclear whether AI reduces task demands, enhances functional use of existing WM, or changes WM capacity itself.

Domain-specific evidence further sharpens this picture. Xiao et al. (2025) compared high- and low-achieving L2 Chinese learners (*N* = 64) in an intensive immersion program and found significant group differences in forward digit span (M = 5.94 vs. 4.80, *p* < 0.05) and backward digit span (M = 5.26 vs. 4.03, *p* < 0.05), with backward span showing the strongest correlation with achievement (r = 0.44, *p* < 0.01). Spatial WM showed no group differences. These findings underscore that verbal rather than spatial WM is most critical for L2 success, a nuance particularly relevant for AI designs that might otherwise target generic “cognitive load reduction” rather than specific verbal and phonological processes.

### 3.2 Affordances

Alongside these challenges, the corpus highlighted a rich set of WM-related affordances that interactive and AI-mediated environments can offer when appropriately designed.

In the Interactive Corpus, well-designed interactive and multimodal features supported richer encoding by providing complementary visual and auditory cues, thereby reducing reliance on a single channel and enabling more robust mental representations. Multimedia glosses, captioned video, and synchronized audio-text presentations were particularly effective when they minimized split attention and coordinated information presentation (Teng, 2019, 2023).

Adaptive and personalized feedback emerged as another important affordance. Even relatively simple pre-AI adaptive mechanisms—such as choice of difficulty level, branching based on performance, or targeted hints—helped learners align task demands with their WM capacity and fostered a sense of competence and autonomy (Schumacher and Ifenthaler, 2018).

Interactive environments also provided substantial opportunities for collaborative and social learning, enabling learners to share the cognitive burden of complex tasks through peer explanation, joint problem solving, and collaborative writing or reading (Apps et al., 2019; Johnson et al., 2014). In these contexts, WM demands could be distributed across group members, mitigating individual capacity limitations.

Multiple studies indicated that thoughtfully designed environments enhanced attention and engagement via gamification and motivational elements, which protected WM by reducing off-task behavior and supporting sustained focus (Chan et al., 2022; Parmaxi and Zaphiris, 2017). Similarly, tasks that explicitly activated prior knowledge and schema facilitated chunking and integration of new information, thereby improving WM efficiency (Huffman and Hahn, 2017; Lin et al., 2022).

Finally, flexible and adaptive learning opportunities—such as self-paced modules, on-demand replay, and ubiquitous access via mobile devices—allowed learners to regulate their own exposure and rehearsal schedules, reducing time pressure and enabling more strategic use of WM resources (Hwang et al., 2012; Zhonggen et al., 2019).

The AI-Mediated Cluster builds on these affordances and introduces qualitatively new possibilities. One of the most significant innovations is dynamic biometric-based adaptation. Traditional systems adjusted difficulty primarily after learners’ correctness or response time indicated struggle. In contrast, Yuan (2025) implemented an AI-enhanced adaptive reading platform (Smart Sparrow) that used eye-tracking (Tobii Pro X3-120) and physiological monitoring to adjust text difficulty for 300 Chinese EFL learners over 12 weeks. The AI-adaptive group outperformed a non-adaptive online reading control on PET reading comprehension (posttest M = 82.6 vs. 70.2; *F*(1, 298) = 118.34, *p* < 0.001, η^2^ = 0.28) and reported significantly lower overall cognitive load on the Differentiated Cognitive Load Questionnaire (M = 3.1 vs. 4.0; F(1, 298) = 61.45, *p* < 0.001, η^2^ = 0.17). Eye-tracking indices—shorter fixation durations (250 ms vs. 285 ms), fewer regressions per 100 words (4.5 vs. 6.5), and longer saccades (8.2 vs. 7.1 characters)—indicated more efficient processing. In effect, the platform operated as an external regulator of WM demands, adjusting difficulty during processing rather than waiting until errors accumulated.

Meta-analytic evidence suggests a further affordance in the form of compensation for developmental WM limitations. Synthesizing 15 AI-assisted L2 learning studies (N = 2,156), Xu et al. (2025) reported a large overall effect of AI-assisted instruction (g = 1.167), with especially strong effects for vocabulary (d = 2.210) and receptive skills (d = 2.011). Notably, K–12 learners showed descriptively larger gains (d = 1.445, 95% CI [1.377, 1.512]) than college students (d = 0.988, 95% CI [0.934, 1.042]), a pattern consistent with the idea that AI’s adaptive pacing and scaffolding can compensate for still-developing WM. Although this difference did not reach conventional significance (*p* = 0.066), it suggests that AI may reduce performance gaps associated with lower WM, aligning with theoretical calls for AI-enabled Aptitude–Treatment Interaction designs (Sana and Fenesi, 2025).

AI systems also enable scaffolded noticing through AI-mediated data-driven learning (DDL). Traditional DDL requires learners to manually query corpora, scan concordance lines, and infer patterns, all of which impose heavy WM demands. Esfandiari and Allaf-Akbary (2024) compared three conditions for 93 advanced Iranian EFL learners: AI-mediated intelligent DDL using Microsoft Copilot (GPT-4-based), traditional corpus-based DDL using AntConc, and conventional instruction. The AI-mediated group dramatically outperformed both comparison groups on the realization of interactional metadiscourse markers in writing (posttest M = 16.16 vs. 12.00 vs. 11.38). ANCOVA revealed a very large group effect (*F*(2, 92) = 168.04, *p* < 0.001, partial η^2^ ≈ 0.79), with Hedges’ g ≈ 3.39 for AI versus control. While WM was not directly measured, the authors argued—drawing on Schmidt’s noticing hypothesis—that Copilot’s ability to locate, cluster, and explain usage patterns reduced the WM-intensive search burden inherent in manual corpus exploration, freeing WM resources for higher-level noticing and abstraction. This type of AI-facilitated pattern highlighting was not available in the traditional CALL tools represented in the Interactive Corpus.

AI-driven multimodality also appears to refine the affordances of multimedia input (Cosentino and Giannakos, 2023). Yu’s (2025) VR–AR study suggested that when channel complexity is held within the empirically defined range of three to four concurrent sources, dual-channel processing is optimized and retention improves by roughly one quarter relative to less complex conditions. At the same time, individual differences in sensory processing moderated the benefits of multimodal AI environments (*β* = 0.31, *p* = 0.007), indicating that optimal multimodal orchestration must be tuned not only to general WM limits but also to learner-specific processing profiles.

Findings from AI-adjacent multimedia research clarify indirect WM pathways. Teng (2024), in a captioned video study without AI, found that WM (Reading Span Task) did not directly predict incidental vocabulary learning but significantly predicted English proficiency (*γ* = 0.372, *p* = 0.002), which in turn predicted vocabulary gains for comedy and educational video genres (*β* ≈ 0.544–0.551). This mediation pattern suggests that WM may support technology-mediated learning indirectly by supporting proficiency development, which then facilitates efficient processing of rich input. For AI design, this implies that systems that accelerate proficiency may indirectly enhance the functional contribution of WM, even if WM is not targeted as an outcome.

Taken together, the corpus portrays a transition from environments that mainly test the limits of WM (Interactive Corpus) to systems that can regulate, redistribute, and sometimes compensate for WM demands (AI-Mediated Cluster), albeit with important unresolved questions about how WM itself is affected (Table 4).

**Table 4:** Affordances identified across technological eras.

Affordance	Operational definition/description	Evidence in interactive corpus (Study IDS)	Evidence in AI-mediated cluster (Study IDs)	Representative studies	Notes on mechanisms relevant to WM/cognitive load
Multimodal support	Provision of complementary visual and auditory cues that enable dual-channel encoding, reducing reliance on a single WM subsystem	Teng (2019, 2023); Kozan et al. (2015); Lusk et al. (2009); Türk and Erçetin (2014); Varol and Erçetin (2021)	Yu (2025)—AI-orchestrated VR–AR with empirically bounded channel limits (3–4 sources optimal)	Teng (2023); Yu (2025)	Engages phonological loop and visuospatial sketchpad in parallel; reduces split attention when modalities are coordinated; AI enables dynamic channel orchestration within empirically defined limits
Adaptive feedback	System-provided feedback that adjusts difficulty, pacing, or scaffolding based on learner performance or state	Schumacher and Ifenthaler (2018); Hwang et al. (2012); Hong et al. (2021)	Yuan (2025)—biometric-adaptive platform using eye-tracking and physiological monitoring for preemptive regulation	Yuan (2025); Schumacher and Ifenthaler (2018)	Aligns task demands with available WM capacity; AI extends from reactive (post-error) to preemptive (during-processing) regulation; reduces extraneous load by preventing overload before errors accumulate
Collaborative/social learning	Opportunities for peer interaction that distribute cognitive burden across group members	Apps et al. (2019); Johnson et al. (2014)	*Evidence gap*: No AI-mediated studies in the cluster examined AI-facilitated collaboration with WM/cognitive load measures	Apps et al. (2019)	Distributes WM demands across participants; enables joint problem-solving and shared explanation; AI potential for intelligent grouping and collaborative scaffolding remains untested
Gamification/engagement	Game-like elements (points, levels, challenges) that sustain attention and reduce off-task behavior	Chan et al. (2022); Parmaxi and Zaphiris (2017); Weissheimer et al. (2019); Hong et al. (2021)	*Evidence gap*: AI-mediated cluster did not include gamified AI interventions with WM/cognitive load assessment	Chan et al. (2022); Hong et al. (2021)	Protects WM by reducing distraction and supporting sustained focus; moderate arousal enhances engagement without exceeding WM capacity; AI-adaptive gamification with WM measures is an untested design space
Schema/Prior Knowledge Activation	Instructional features that explicitly connect new information to existing knowledge structures	Huffman and Hahn (2017); Lin et al. (2022)	*Evidence gap*: AI-mediated studies did not explicitly examine schema activation mechanisms	Huffman and Hahn (2017)	Facilitates chunking and integration, improving WM efficiency; reduces intrinsic load by leveraging long-term memory; AI potential for personalized schema priming based on learner knowledge profiles is unexplored
Flexible/Self-Paced Learning	Learner control over pacing, replay, and access timing that enables strategic WM resource management	Hwang et al. (2012); Zhonggen et al. (2019); Kukulska-Hulme (2019)	Yuan (2025)—AI dynamically adjusts pacing based on real-time biometric indicators	Yuan (2025); Zhonggen et al. (2019)	Reduces time pressure; allows rehearsal and consolidation; AI transforms flexibility from learner-controlled to system-optimized based on cognitive state indicators
Biometric-Based Adaptation	Real-time adjustment of task parameters based on physiological indicators (eye-tracking, arousal)	*Not present*: Pre-AI systems lacked biometric integration for WM-relevant adaptation	Yuan (2025)—eye-tracking indices (fixation duration, regressions, saccade length) used to regulate text difficulty	Yuan (2025)	Enables preemptive load regulation during processing; shorter fixations and fewer regressions indicate more efficient WM utilization; represents qualitative shift from reactive to anticipatory support
Cognitive Load Redistribution	Shifting cognitive demands from lower-level encoding to higher-order evaluation and integration	*Not applicable*: Interactive tools reduced load but did not systematically redistribute across WM subsystems	Fan and Yao (2025)—CL-AI-L2W scale demonstrates redistribution from Authorial Core Processing (M = 3.48) to Critical Evaluation (M = 4.81), Prompt Management (M = 4.55), and Integrative Synthesis (M = 4.40)	Fan and Yao (2025)	Offloads phonological loop demands (lexical retrieval, grammatical encoding); increases central-executive demands (monitoring, verification, integration); learners lacking metacognitive strategies may experience net increase in perceived difficulty
WM Compensation for Developmental Differences	AI support that disproportionately benefits learners with lower or still-developing WM capacity	*Limited evidence*: Interactive studies showed WM as predictor but not compensation target	Xu et al. (2025) meta-analysis—K–12 learners (d = 1.445) showed descriptively larger gains than college students (d = 0.988), consistent with compensatory function	Xu et al. (2025)	AI adaptive pacing may reduce performance gaps associated with lower WM; aligns with ATI framework predictions; direct WM measurement needed to confirm compensation vs. demand reduction mechanisms
Scaffolded Noticing via AI-DDL	AI automation of corpus search and pattern highlighting that frees WM for higher-order abstraction	*Partial precedent*: Traditional DDL (e.g., AntConc) required WM-intensive manual search	Esfandiari and Allaf-Akbary (2024)—AI-mediated DDL (Microsoft Copilot) dramatically outperformed traditional DDL (Hedges’ g ≈ 3.39 vs. control)	Esfandiari and Allaf-Akbary (2024)	Reduces WM burden of concordancing and pattern identification; theorized to free resources for noticing (Schmidt’s hypothesis) and internalization; AI locates, clusters, and explains usage patterns automatically

WM, working memory; DDL, data-driven learning; ATI, aptitude–treatment interaction; CL-AI-L2W, Cognitive Load Scale for AI-Assisted L2 Writing. Evidence gaps are explicitly marked where affordances lack empirical examination with WM or cognitive load measures in the respective corpus. Effect sizes and descriptive statistics are reported where available from primary studies. Mechanisms column indicates specific WM subsystems engaged (phonological loop, visuospatial sketchpad, central executive) and load types affected (intrinsic, extraneous, germane; Table 5).

**Table 5:** Design principles to minimize cognitive overload.

Principle (actionable)	Theoretical rationale (WM/cognitive load linkage)	Supporting evidence	Application in interactive environments	Application in AI-mediated environments	Evidence strength/validation status
Limit concurrent information channels to 3–4 sources	Exceeding channel capacity overwhelms the central executive and depletes phonological loop and visuospatial sketchpad resources simultaneously, consistent with Cowan's (2001) magical number 4 limit for short-term memory chunks	Yu (2025): Inverted-U relationship with optimal retention at 3–4 channels (R^2^ = 0.41); 61% reported overload beyond threshold; *β* = −0.53 for overload–retention relationship	Coordinate text, audio, and images to avoid split attention; minimize simultaneous animations and hyperlinks (Kozan et al., 2015; Lusk et al., 2009)	AI systems should dynamically orchestrate multimodal input within empirically bounded limits; VR–AR platforms must monitor and cap concurrent sensory streams	Supported by experimental RCT (Yu, 2025) and foundational WM theory (Cowan, 2001); replicated across multimedia studies
Implement preemptive rather than reactive difficulty adjustment	Reactive adaptation (post-error) allows cognitive overload to accumulate before intervention; preemptive regulation maintains WM load within capacity limits during processing, preventing error cascades	Yuan (2025): Biometric-adaptive reading reduced cognitive load (M = 3.1 vs. 4.0; η^2^ = 0.17) and improved comprehension (η^2^ = 0.28) with eye-tracking-based preemptive adjustment	Limited to post-performance branching; difficulty adjusted after errors or slow response times accumulate (Schumacher and Ifenthaler, 2018)	AI platforms can use real-time biometric indicators (eye-tracking, physiological monitoring) to adjust text complexity before overload manifests	Supported by RCT with biometric validation (Yuan, 2025); theoretical alignment with CLT—needs replication across modalities
Offload lower-level encoding tasks to free resources for higher-order processing	Automating routine phonological loop demands (lexical retrieval, syntactic assembly) frees central-executive resources for comprehension, integration, and evaluation (Sweller et al., 2019)	Esfandiari and Allaf-Akbary (2024): AI-mediated DDL outperformed traditional DDL (Hedges’ g ≈ 3.39) by offloading search burden; Fan and Yao (2025): Authorial Core Processing showed lowest load (M = 3.48)	Interactive glosses reduce lookup burden (Türk and Erçetin, 2014); captions offload auditory decoding (Teng, 2019, 2023)	Generative AI chatbots automate pattern search and lexical retrieval; AI-assisted writing reduces encoding demands while freeing WM for synthesis	Supported by multiple RCTs and scale validation; very large effect sizes for AI-DDL; consistent with noticing hypothesis
Coordinate modalities to support dual-channel processing without redundancy	Presenting complementary (not identical) information across visual and auditory channels leverages both WM subsystems; redundant presentation wastes capacity (Mayer, 2014; Mutlu-Bayraktar et al., 2019)	Teng (2019, 2023): Captioned video enhanced comprehension when captions complemented rather than duplicated audio; Varol and Erçetin (2021): Gloss coordination improved outcomes	Synchronize audio-text presentations; avoid simultaneous identical captions and narration; use glosses that add rather than repeat information	AI can personalize channel combinations based on learner processing profiles; dynamic modality switching based on task phase and learner state	Supported by multiple quasi-experimental and correlational studies; consistent with multimedia learning principles; AI personalization is proposed—needs direct WM validation
Scaffold metacognitive evaluation skills when AI redistributes load	AI offloads encoding but increases central-executive demands for critical evaluation and integration; learners lacking metacognitive strategies may experience net cognitive burden increase (Fan and Yao, 2025; Li, 2023)	Fan and Yao (2025): Critical Evaluation (M = 4.81), Prompt Management (M = 4.55), and Integrative Synthesis (M = 4.40) showed highest load in AI-assisted writing; CL-AI-L2W *α* = 0.94	Not directly applicable; pre-AI tools did not systematically redistribute load toward evaluation	Explicitly teach prompt formulation, output verification, and AI–human integration skills before deploying AI writing/DDL tools; provide metacognitive scaffolding	Supported by validated scale development (Fan and Yao, 2025); theoretical support from CLT redistribution literature; pedagogical intervention studies needed
Grade task complexity to match proficiency and WM capacity	Intrinsic cognitive load rises with linguistic complexity; when unscaffolded, this saturates WM and produces floor effects for lower-proficiency learners (Sweller et al., 2019)	Denhovska et al. (2016); Makransky et al. (2016): Lower-proficiency learners showed steeper WM-related performance decrements; Xiao et al. (2025): Verbal WM (backward span) strongly predicted L2 achievement (r = 0.44)	Provide difficulty selection options; sequence tasks from simple to complex; offer optional scaffolding for complex morphosyntax	AI systems can continuously adjust linguistic complexity based on real-time performance and, potentially, WM indicators; K–12 learners may benefit disproportionately (Xu et al., 2025: d = 1.445 vs. 0.988)	Supported by multiple correlational and quasi-experimental studies; meta-analytic evidence for developmental differences; ATI validation with direct WM measures needed
Minimize extraneous technical and interface demands	Technical difficulties (slow connections, unintuitive interfaces, system crashes) impose extraneous cognitive load, diverting WM from linguistic processing (Golonka et al., 2014; Hwang et al., 2013)	Golonka et al. (2014); Hwang et al. (2013); Sweller et al. (2019): Technical issues correlated with reduced learning outcomes and increased frustration	Design intuitive interfaces; ensure reliable connectivity; minimize navigation complexity; provide clear instructions	AI platforms must maintain seamless performance; avoid latency-induced attention shifts; ensure AI response consistency to prevent troubleshooting demands	Supported by multiple observational and correlational studies; consistent with extraneous load reduction principles; direct WM impact studies limited
Support distributed cognition through collaborative structures	Distributing task demands across group members reduces individual WM burden; peer explanation and joint problem-solving leverage collective cognitive resources (Apps et al., 2019; Johnson et al., 2014)	Apps et al. (2019); Johnson et al. (2014): Collaborative structures improved outcomes in WM-demanding online tasks through shared explanation and distributed processing	Incorporate peer discussion, collaborative writing, and joint problem-solving activities; structure group roles to distribute cognitive load	AI could facilitate intelligent grouping based on complementary WM profiles or provide AI-mediated collaborative scaffolding; this design space remains untested	Supported by quasi-experimental studies in interactive contexts; theoretical alignment with distributed cognition; AI-facilitated collaboration with WM measures is an evidence gap
Activate prior knowledge and schema before introducing new material	Schema activation facilitates chunking, reducing intrinsic load by allowing new information to integrate with existing long-term memory structures (Huffman and Hahn, 2017; Sweller et al., 2019)	Huffman and Hahn (2017); Lin et al. (2022): Prior knowledge activation improved WM efficiency and enhanced retention in technology-mediated tasks	Use advance organizers; preview key vocabulary; connect new content to familiar concepts before complex multimedia exposure	AI systems could personalize schema priming based on learner knowledge profiles inferred from interaction history; this potential is currently unexplored	Supported by experimental and quasi-experimental studies; theoretically grounded in schema theory and CLT; AI-personalized activation is proposed—needs empirical testing
Incorporate direct WM assessment for adaptive calibration	Without direct WM measurement, systems cannot distinguish whether interventions reduce task demands, improve resource utilization, or enhance capacity itself; proxies conflate distinct mechanisms	Chalmers et al. (2021); Goh and Aryadoust (2025); Zhang and Aubrey (2024): Systematic reviews document measurement gap; Teng (2024): WM–learning relationships may be mediated by proficiency	Occasional inclusion of span tasks for research purposes; individual differences typically unmeasured in routine instruction	AI systems should integrate validated WM assessments (e.g., automated span tasks) for real-time adaptive calibration and APT-based personalization	Proposed based on measurement gap analysis; theoretically critical for causal inference; no current AI implementations include validated WM assessment

WM, working memory; CLT, Cognitive Load Theory; DDL, data-driven learning; ATI, aptitude–treatment interaction; RCT, randomized controlled trial; CL-AI-L2W, Cognitive Load Scale for AI-Assisted L2 Writing. Evidence strength is categorized as: “Supported by multiple experimental studies” (convergent evidence from ≥2 RCTs or quasi-experiments with consistent findings), “Supported by experimental/quasi-experimental study” (single well-designed study), “Supported by correlational/observational studies” (non-experimental evidence), “Proposed—needs direct WM validation” (theoretically grounded but lacking empirical WM assessment), or “Evidence gap” (no studies examining the principle with WM/cognitive load measures in the specified environment). Effect sizes and statistical parameters are reported where available from primary studies.

### 3.3 Adopted instructional tools and comparative overview

The Interactive Corpus employed a range of tools to create interactive language learning environments. Online platforms were the most common, appearing in approximately 48% of the 27 studies. CALL software was used in about 24% of studies, mobile applications in 16%, and early non-AI virtual reality environments in 12% (Makransky et al., 2016). All interactive tools integrated some combination of multimedia features—videos, images, animations—to support WM by providing redundant cues and multiple input channels. Approximately three quarters of the studies incorporated gamification elements or adaptive feedback mechanisms to maintain attention and engagement, and more than one third provided structured opportunities for peer collaboration, while roughly two fifths used individualized feedback based on learners’ performance trajectories.

The AI-Mediated Cluster introduced a qualitatively different family of tools. These included generative AI chatbots such as DeepSeek V3.1 for AI-assisted writing (Fan and Yao, 2025) and Microsoft Copilot for intelligent DDL (Esfandiari and Allaf-Akbary, 2024); biometric-adaptive reading platforms such as Smart Sparrow augmented with eye-tracking and physiological monitoring (Yuan, 2025); and AI-orchestrated VR–AR systems combining Unity, Oculus Quest 2, and Google MediaPipe with ChatGPT-4 for multimodal vocabulary learning (Yu, 2025). Complementary reviews documented emerging use of intelligent personal assistants and spoken dialogue systems for listening and speaking practice (Goh and Aryadoust, 2025; Xu, 2025).

Conceptually, this shift can be summarized as a movement from pre-scripted, reactive tools to dynamic, dialogic cognitive partners. Feedback systems move from pre-programmed corrective responses to open-ended generative explanations; adaptation mechanisms move from performance-based difficulty adjustment to biometric-driven, real-time regulation; multimodal delivery moves from static multimedia to embodied VR–AR experiences orchestrated by LLMs; and personalization moves from predetermined learning paths to dynamic prompt–response dialogue.

At the same time, a methodological divergence is evident. Several Interactive Corpus studies occasionally incorporated direct WM measures (e.g., digit spans, reading span tasks), enabling explicit WM–performance analyses. None of the four AI-mediated primary studies, nor the broader AI-in-practice literature summarized in recent reviews, included validated WM instruments. Instead, AI studies relied on self-report cognitive load scales and process indicators as proxies. This divergence complicates direct comparison of WM effects across eras and contributes to the measurement paradox that emerges from the broader synthesis.

In summary, the comparative analysis reveals both continuities and transformations. Cognitive overload remains a central concern, but AI research now specifies empirical boundaries such as Yu’s (2025) three- to four-channel optimum. Individual differences in WM continue to shape technology effectiveness, yet AI-mediated environments show potential to compensate for lower or immature WM through adaptive pacing and targeted support (Xu et al., 2025). Multimodal presentation remains beneficial, though AI allows dynamic, learner-sensitive orchestration rather than static design. Perhaps most critically, the locus of cognitive challenge has shifted: from managing dense input and technical distractions in the Interactive Corpus to managing higher-order evaluative and integrative demands in the AI-Mediated Cluster. At the same time, direct measurement of WM has declined just as AI research relies more heavily on WM-based explanations, leaving open key questions about whether AI primarily changes task demands, functional WM use, or WM capacity itself—questions that subsequent sections take up in the Discussion and Future Directions.

## 4 Discussion

Returning to the three research questions, this comparative synthesis illuminates a fundamental shift in the architecture of technology-enhanced language learning—from a hermeneutics of accommodation to a hermeneutics of co-regulation. Regarding RQ1 (design guidelines), the Interactive Corpus demonstrates that multimodal coordination, adaptive pacing, and schema activation support WM efficiency, whereas information density, technical disruptions, and unscaffolded complexity overload capacity. Regarding RQ2 (WM × AI affordances interactions), AI-mediated environments exhibit qualitatively distinct interaction patterns: rather than simply reducing load, they redistribute cognitive demands from encoding (phonological loop) to evaluation and integration (central executive), as evidenced by the CL-AI-L2W factor structure and biometric-adaptive process data. Regarding RQ3 (boundary conditions and unintended consequences), the review identifies empirical channel limits (3–4 concurrent sources in VR–AR), the measurement paradox (increasing theoretical invocation but decreasing direct assessment of WM), and the risk that load redistribution may disadvantage learners lacking metacognitive strategies, as well as potential over-scaffolding (i.e., overprotection) with downstream effects on metacognitive monitoring and executive-skill development.

In the pre-AI corpus, working memory was treated as a finite, static buffer: an invariant biological constraint around which instructional design had to be carefully engineered. Technologies were expected to adapt; cognition was assumed to be fixed. The design mandate was therefore to manage information density, sequencing, and modality so that task demands remained within known working-memory limits, consistent with the core tenets of Cognitive Load Theory and multimedia learning frameworks (Mayer, 2014; Sweller, 2020).

The emergence of AI-mediated environments, particularly those integrating generative AI and biometric sensing, destabilizes this arrangement. The unit of analysis is no longer the solitary learner but a dynamically coupled human–AI system. In this regulatory model, working-memory demands are not simply “respected” but actively monitored, redistributed, and, at least in principle, shaped over time. Biometric-adaptive systems that adjust text complexity in real time based on physiological markers of arousal and effort exemplify this shift from reactive accommodation to preemptive regulation (Yuan, 2025). Meta-analytic evidence that K–12 learners, whose working memory is still developing, can achieve gains comparable to or larger than adults when supported by AI pacing and feedback suggests that AI may function as a compensatory prosthesis rather than merely a gentler delivery channel (Xu et al., 2025). In this sense, AI is not simply a scaffold resting on a fixed cognitive foundation; it is an active participant in the regulation of task demands and cognitive resources.

A central theme emerging from this comparison is the move from load reduction to load redistribution. Traditional interactive environments, informed by Cognitive Load Theory, sought to lower overall cognitive load by minimizing extraneous demands and managing intrinsic complexity. AI-mediated environments, by contrast, alter the structure of the load rather than its sheer volume. Generative AI in L2 writing offloads lower-level encoding demands—such as lexical retrieval and syntactic assembly—that classically tax the phonological loop, while substantially increasing demands on central-executive processes: prompt formulation, output verification, and integrative synthesis of human and machine contributions (Fan and Yao, 2025). The factor structure and mean profiles of the CL-AI-L2W scale, with relatively lower load on authorial core processing and higher load on evaluation and management, empirically capture this redistribution. Similarly, AI-mediated data-driven learning automates manual pattern search and concordancing, freeing cognitive resources for higher-order noticing and internalization, as reflected in the substantial performance advantages of AI-supported DDL over traditional corpus-based tasks (Esfandiari and Allaf-Akbary, 2024).

This redistribution is double-edged. For learners with robust executive control and metacognitive strategies, AI may indeed create a cognitive surplus—that is, freed working memory capacity available for higher-order processing when routine demands are offloaded (Sweller et al., 2019)—by removing routine encoding burdens. For learners with fragile central-executive resources or limited experience in critical evaluation, the same tools may intensify perceived difficulty: the effort saved in generating language is immediately reallocated to monitoring, checking, and reconciling AI output with task requirements and personal goals. The benefits of AI are therefore conditional, not automatic, and hinge on learners’ preparedness for the new forms of cognitive work that AI-mediated tasks demand.

The review also exposes a deeper epistemic problem: the Measurement Paradox. As the field’s theoretical discourse on AI and cognition grows more sophisticated—foregrounding working memory, cognitive load, and aptitude–treatment interactions—the direct measurement of working memory has receded. Many of the pre-AI studies included validated span tasks or closely related measures, enabling explicit tests of how working-memory capacity constrained or enabled learning. In contrast, the AI-mediated intervention studies, despite their claims about compensation and optimization, rely almost exclusively on subjective cognitive load ratings, proficiency scores, or process indicators as proxies for working memory. Across multiple systematic reviews, working memory is repeatedly invoked as central to aptitude and AI-mediated learning, yet rarely measured with psychometric rigor (Chalmers et al., 2021; Goh and Aryadoust, 2025; Sana and Fenesi, 2025; Zhang and Aubrey, 2024; Xu, 2025). Teng’s (2024) finding that working memory influences vocabulary learning indirectly via proficiency illustrates the complexity of these relationships and underscores that nuanced, multi-step pathways cannot be inferred from load ratings alone.

This reliance on proxies creates a causal black box. When an AI intervention is associated with lower self-reported load and better outcomes, we cannot determine whether the AI simplified the task environment, improved learners’ allocation of existing working-memory resources, or contributed to durable changes in capacity. These mechanisms are conceptually distinct and carry very different implications for design, equity, and long-term development, yet they are empirically conflated when working memory is left unmeasured.

Domain-specific and neurocognitive evidence in the corpus further complicates the picture but also opens new possibilities. Findings that verbal working-memory measures, particularly backward digit span, differentiate high- and low-achieving L2 learners more strongly than spatial measures suggest that specific subsystems of working memory are especially critical for language learning (Xiao et al., 2025). Reviews of multisensory working-memory training using linguistically rich materials indicate that aspects of working memory may be more plastic than trait models assumed, with gains in capacity and neural efficiency accompanied by transfer to new auditory tasks (Gkintoni et al., 2025). Although these studies are not themselves AI-mediated, they point toward an underexplored design space in which AI could be used not only to compensate for working-memory constraints but also to deliberately train key verbal working-memory processes in ecologically valid language tasks.

Taken together, the present review suggests that the field stands at a crossroads. On one path lies a sophisticated narrative of AI as a co-regulator of cognition that compensates for developmental and individual differences in working memory. On the other lies a persistent methodological reticence to measure working memory directly, leaving fundamental causal questions unanswered. At present, the evidence base is insufficient to adjudicate longer-run developmental trade-offs; a balanced account therefore must specify where AI-mediated support may backfire through over-scaffolding (i.e., overprotection), observable as dependency on AI support, reduced independent strategy use, and reduced transfer under reduced-support conditions, with potential downstream costs for metacognitive monitoring and executive-skill development. These boundary conditions can be tested empirically by incorporating scaffold-fading schedules, explicit reduced-support phases, delayed transfer tasks, and metacognitive/executive outcome measures (e.g., monitoring accuracy, strategy-use indices, and executive-control measures) alongside immediate performance outcomes. Bridging this gap is essential if AI is to be understood—and responsibly deployed—not merely as a convenience layer on existing pedagogies, but as a transformative partner in the orchestration of cognitive effort.

### 4.1 Synthesis by research question: evidence, confidence, and remaining gaps

RQ1 (Design Guidelines): What instructional design features of interactive language learning environments (2010–2024) support working memory efficiency, and which features challenge or overload working memory capacity?

*Interactive Corpus Evidence.* The 27 pre-AI studies consistently demonstrate that multimodal coordination (synchronized audio-visual presentation), adaptive pacing, schema activation, and collaborative task structures support WM efficiency by distributing demands across subsystems and enabling chunking. Conversely, high information density, unscaffolded complexity, technical disruptions, and unregulated multitasking overtax WM capacity, particularly for lower-capacity learners.

*AI-Mediated Cluster Evidence.* The four AI-mediated studies do not directly test traditional design features but confirm that the principles identified in the Interactive Corpus remain operative: Yu (2025) demonstrates that even AI-orchestrated multimodality produces overload when channel complexity exceeds three to four concurrent sources, and Yuan (2025) shows that adaptive pacing—when enhanced with biometric sensing—substantially reduces cognitive load and improves outcomes.

*Confidence and Limits.* Confidence in the design guidelines is moderate to high for the Interactive Corpus, supported by multiple methodologically diverse studies with some direct WM measurement. Confidence for AI contexts is lower because AI-mediated studies did not isolate traditional design features experimentally.

*What Remains Unknown.* Whether specific design features (e.g., schema activation, collaborative scaffolding) interact differently with AI affordances than with pre-AI tools remains untested. Highest-priority empirical test: A factorial experiment comparing schema-priming and collaborative-support manipulations in matched AI-mediated versus non-AI conditions, with direct WM assessment at baseline and post-intervention.

RQ2 (WM × AI Affordances Interactions): How do AI-mediated language learning affordances—including generative chatbots, biometric-adaptive systems, and multimodal VR–AR platforms—interact with working memory processes, and do these interactions differ qualitatively from those observed in traditional interactive environments?

*Interactive Corpus Evidence.* In pre-AI environments, WM was primarily engaged at the input-processing level (phonological loop, visuospatial sketchpad), and individual WM capacity functioned as a moderator of learning outcomes. Higher-capacity learners benefited more from complex multimedia and self-directed tasks; adaptation was reactive and performance-based.

*AI-Mediated Cluster Evidence.* AI affordances produce qualitatively distinct interaction patterns. Fan and Yao's (2025) CL-AI-L2W scale reveals that generative AI shifts the locus of cognitive demand from lower-level encoding (Authorial Core Processing: M = 3.48) to central-executive functions (Critical Evaluation: M = 4.81; Prompt Management: M = 4.55; Integrative Synthesis: M = 4.40). Yuan (2025) demonstrates that biometric-adaptive systems enable preemptive rather than reactive regulation, adjusting difficulty during processing before errors accumulate. Esfandiari and Allaf-Akbary (2024) show that AI-mediated DDL offloads search demands, theoretically freeing WM for higher-order noticing. Meta-analytic evidence (Xu et al., 2025) suggests that AI may compensate for developmental WM limitations, with K–12 learners showing descriptively larger gains than adults.

*Confidence and Limits.* Confidence that AI redistributes load is moderate, supported by validated scale data and effect sizes. However, confidence in the compensation hypothesis is limited because no AI-mediated study directly measured WM capacity; inferences rely on cognitive load proxies and age-based comparisons.

*What Remains Unknown.* Whether AI-mediated load redistribution benefits or disadvantages learners with different WM profiles (high vs. low capacity, verbal vs. spatial strengths) cannot be determined without direct WM assessment. Highest-priority empirical test: An aptitude–treatment interaction study crossing AI condition (generative AI-assisted writing vs. control) with baseline WM capacity (measured via validated span tasks), examining whether AI selectively benefits lower-WM learners or intensifies central-executive demands for all.

RQ3 (Boundary Conditions and Unintended Consequences): What are the empirical boundary conditions of AI-mediated working memory support, and what unintended consequences—including cognitive load redistribution and measurement gaps—emerge from AI integration in language learning?

*Interactive Corpus Evidence.* Pre-AI studies identified general overload thresholds tied to information density and task complexity but did not quantify precise channel limits. Unintended consequences included performance gaps favoring higher-WM learners and anxiety-induced WM impairment under high-stakes or unfamiliar conditions.

*AI-Mediated Cluster Evidence.* Yu (2025) provides the first empirically specified boundary condition: an inverted-U relationship between multimodal channel complexity and retention, with optimal performance at three to four concurrent sources (R^2^ = 0.41) and significant overload beyond this threshold (*β* = −0.53). The redistribution of load from encoding to evaluation (Fan and Yao, 2025) constitutes an unintended consequence: learners lacking metacognitive strategies may experience AI support as cognitively more demanding despite reduced encoding burden. The measurement paradox—whereby WM is increasingly invoked theoretically but decreasingly measured empirically—emerges as a cross-cutting methodological consequence, documented across multiple systematic reviews (Chalmers et al., 2021; Goh and Aryadoust, 2025; Zhang and Aubrey, 2024).

*Confidence and Limits.* Confidence in the three-to-four channel boundary is moderate, based on a single large-scale RCT; replication across populations and modality combinations is needed. Confidence in the load redistribution pattern is moderate to high, given the robust factor structure and fit indices of the CL-AI-L2W scale. Confidence regarding the measurement paradox is high, as it is documented across multiple independent reviews.

*What Remains Unknown.* Whether the channel boundary generalizes beyond VR–AR vocabulary learning to other skills and AI configurations remains untested. Whether load redistribution produces differential effects for learners with varying central-executive capacity is unknown. Highest-priority empirical test: A replication of Yu (2025) with direct WM measurement (especially executive-function tasks) to determine whether individual differences in central-executive capacity moderate the channel-limit threshold and overload effects.

### 4.2 Objective attainment statement

The three research objectives were substantially but incompletely met. Objective 1 (aligned with RQ1) was largely achieved: the review synthesized consistent evidence-based design guidelines for WM-sensitive interactive instruction from the 27-study Interactive Corpus. Objective 2 (aligned with RQ2) was partially achieved: the review identified qualitatively distinct WM × AI interaction patterns (load redistribution, preemptive regulation, potential compensation), but the absence of direct WM measurement in AI studies limits causal conclusions. Objective 3 (aligned with RQ3) was substantially achieved: the review documented specific boundary conditions (channel limits), unintended consequences (load redistribution disadvantaging metacognitively unprepared learners), and the measurement paradox, though the empirical base for AI-specific boundaries remains narrow.

### 4.3 Prioritized gap list

Based on the synthesis, the following empirical gaps are prioritized for future research:

1. Direct WM measurement in AI intervention studies (highest priority). No AI-mediated intervention in the corpus included validated WM tasks. Without such measurement, it is impossible to distinguish whether AI reduces task demands, improves functional WM utilization, or changes WM capacity itself.
2. Aptitude–treatment interaction designs. Studies are needed that cross AI affordances with baseline WM profiles to test compensation hypotheses and identify which learners benefit most (or least) from specific AI configurations.
3. Replication of multimodal channel boundaries. Yu's (2025) three-to-four channel optimum requires replication across diverse learner populations, language skills, and AI-multimodal configurations.
4. Central-executive load and metacognitive preparedness. Research should examine whether explicit metacognitive training mitigates the increased evaluative demands associated with generative AI use.
5. Longitudinal tracking of WM dynamics. Studies are needed to determine whether sustained AI-mediated instruction affects WM capacity over time or primarily operates through demand reduction.
6. Geographical and linguistic diversification. The AI-mediated evidence base is concentrated in East Asian EFL contexts; replication in other linguistic, cultural, and institutional settings is essential for generalizability.

The paradigm shift from static accommodation to dynamic co-regulation carries far-reaching implications for instructional design, pedagogy, and research.

For design, the central task moves from minimizing load in the abstract to optimizing the alignment between task demands and learners’ evolving cognitive profiles. AI-mediated systems should be conceived as adaptive regulators that can detect signs of overload or underload and adjust pacing, complexity, and modality in real time. Such systems need to distinguish between supportive compensation and overprotection, providing scaffolding that is gradually faded rather than indefinitely maintained. Design choices that automate lower-level processes must be weighed against the risk of eroding opportunities for productive struggle and desirable difficulty. To translate these implications into implementable steps, an evidence-informed checklist for designers and instructors is provided below.

### 4.4 Practical guidelines for designers and instructors

1. Manage information density and enforce channel limits by keeping concurrent information streams to three to four at most and using progressive disclosure and chunking. Rationale: Limiting extraneous load prevents working memory saturation and aligns with observed multimedia overload and empirically bounded multimodal thresholds (Lusk et al., 2009; Kozan et al., 2015; Yu, 2025; Cowan, 2001).
2. Sequence and coordinate modalities to prevent split attention (present core input first, then add complementary cues; avoid redundant text-audio duplication). Rationale: Coordinated multimodal timing supports dual-channel processing and improves comprehension under working memory constraints (Mayer, 2014; Türk and Erçetin, 2014; Kozan et al., 2015).
3. Scaffold strategically, then fade supports based on performance signals (start with guided prompts/worked examples; progressively withdraw assistance as accuracy stabilizes). Rationale: Research-informed recommendation: scaffolding can externalize interim processing to reduce working memory demands, while fading preserves desirable difficulty and independent executive control (Schumacher and Ifenthaler, 2018; Sana and Fenesi, 2025).
4. Implement adaptive pacing and just-in-time feedback (enable pause/replay/stepwise reveal; deliver concise feedback at the point of error or hesitation). Rationale: Adaptive pacing and timely feedback regulate cognitive load and have been associated with improved comprehension alongside reduced load in adaptive learning implementations (Hwang et al., 2012; Yuan, 2025).
5. Use biometric or high-quality behavioral proxies to tune cognitive load where feasible and ethically governed (e.g., eye-tracking indices, response latency, revision traces) with opt-in consent and data minimization. Rationale: Research-informed recommendation: real-time proxy signals can enable preemptive regulation of processing demands, but current language-learning evidence is limited to a small number of implementations and requires privacy and fairness safeguards (Yuan, 2025; Cosentino and Giannakos, 2023; Silva Barbosa et al., 2023).
6. Teach metacognitive self-regulation for AI-supported tasks (plan prompts, verify AI outputs, and justify revisions using checklists and reflection routines). Rationale: AI assistance can shift load toward central-executive evaluation and integration, so explicit metacognitive instruction supports effective working memory allocation and reduces uncritical reliance (Fan and Yao, 2025; Lee, 2021; Goh and Aryadoust, 2025).

Pedagogically, AI integration demands a new emphasis on metacognitive acculturation. If AI shifts the burden from encoding to evaluation, learners must be explicitly taught how to manage this new cognitive landscape. Instruction needs to foreground skills such as structuring effective prompts, monitoring AI outputs for accuracy and relevance, and integrating external suggestions into coherent personal representations. Classroom assessment should, in turn, move beyond evaluating products generated in isolation to examining how learners orchestrate human–AI interaction over time—for example, by analyzing prompt sequences, revision histories, and justification of decisions in AI-supported tasks.

Methodologically, the field must recalibrate its evidentiary standards. Subjective cognitive load measures are insufficient on their own to sustain claims about compensation, optimization, or training effects. A more rigorous paradigm requires the routine inclusion of direct working-memory assessments, the integration of behavioral and process-tracing data, and the systematic testing of aptitude–treatment interactions. Without such shifts, the discourse on AI and working memory will remain largely speculative, even as the technologies themselves become more pervasive.

### 4.5 Integrating direct working memory assessment in AI-mediated research

#### 4.5.1 Instruments and implementation guidelines

To address the persistent absence of direct WM measurement documented across the AI-mediated literature, this section provides concrete guidance for researchers seeking to incorporate validated WM instruments into AI-assisted language learning studies. The recommendations distinguish between direct psychometric WM assessment and behavioral proxy indicators, and offer practical implementation strategies for embedding WM measurement within AI-mediated research designs.

#### 4.5.2 Direct WM assessment versus behavioral proxies

A fundamental distinction must be drawn between direct WM assessment—validated psychometric tasks that isolate WM constructs through controlled stimuli and standardized administration—and behavioral proxy indicators derived from learner interaction logs, response latencies, and eye-tracking metrics. Direct assessment provides construct-valid measures of WM capacity, updating efficiency, or subsystem-specific functioning, enabling causal inference about whether AI interventions affect WM resources, utilization, or capacity itself. Behavioral proxies, by contrast, reflect processing patterns that may be influenced by WM but also by motivation, familiarity, strategy use, and task design. While proxies offer ecological validity and continuous monitoring capability, they cannot substitute for direct measurement when the research question concerns WM mechanisms. Robust AI-mediated research designs should therefore combine direct WM assessment (for construct validity and individual-difference analysis) with behavioral proxies (for process-level insight and real-time monitoring), triangulating findings across measurement modalities.

#### 4.5.3 Curated working memory tasks for computer and Mobile delivery

Table 6 presents a curated set of validated WM tasks suitable for computerized or mobile administration in AI-mediated language learning research. For each task, the table specifies the construct targeted (capacity, updating, or subsystem-specific functioning), administration mode, scoring procedures, feasibility for embedding in AI platforms, minimum psychometric and reporting requirements, and practical implementation guidance including timing burden and practice-effect mitigation strategies.

**Table 6:** Validated working memory tasks for AI-mediated language learning research.

Task	Construct targeted	Administration mode	Scoring	Feasibility for AI platform integration	Psychometric requirements	Implementation guidance
Operation span (OSPAN)	Central executive capacity; complex span	Computer/tablet; 15–20 min; requires keyboard or touch response	Partial-credit unit scoring (sum of correctly recalled items in correct serial position); absolute scoring also reported	High: Can be administered as pre/post module; automated scoring feasible; validated computerized versions available (Unsworth et al., 2005)	Report internal consistency (α ≥ 0.70); test–retest reliability if repeated; cite normative data; report language of stimuli	Use parallel forms or alternate stimulus sets for pre/post to mitigate practice effects; allow one practice trial block; administer in quiet conditions; timing: ~20 min total
Reading span task (RST)	Verbal WM capacity; phonological loop + central executive	Computer/tablet; 15–25 min; requires sentence verification and letter/word recall	Partial-credit scoring; processing accuracy reported separately	High: Well-suited for L2 research; sentence stimuli can be adapted to target language proficiency; automated administration validated	α ≥ 0.70; report both storage and processing accuracy; language-appropriate sentence norming required	Critical for L2 contexts: use proficiency-appropriate sentences to avoid floor/ceiling effects; provide parallel forms; ~20–25 min total including instructions
Backward digit span	Verbal WM capacity; phonological loop + manipulation	Computer/mobile; 5–10 min; audio or visual digit presentation with typed/spoken response	Longest sequence correctly recalled; total correct trials	Very High: Brief; minimal technical requirements; easily embedded as “micro-assessment” between AI tasks	Report span score and total correct; internal consistency via split-half; cite WAIS-IV or equivalent norms	Suitable for intermittent administration (every 2–3 sessions); use parallel digit sequences; ~5–7 min; low practice effects with adequate intervals
N-back (verbal)	Updating; central executive	Computer/tablet; 10–15 min; continuous stimulus presentation with match/non-match response	d′ (sensitivity); accuracy; reaction time	Moderate-High: Requires precise timing control; gamified versions available (Scharinger et al., 2023); suitable for separate assessment module	Report d′, accuracy, and RT; specify n-level (typically 2-back for adequate difficulty); internal consistency via split-half	Higher cognitive demand may induce fatigue; administer at session start; 2-back recommended for most populations; ~12–15 min; parallel stimulus sets available
Corsi block-tapping (backward)	Visuospatial WM capacity	Tablet (touch-based); 5–10 min; sequential block highlighting with reverse-order response	Longest sequence correctly recalled; total correct	High: Touch-screen administration well-validated; suitable for cross-linguistic samples (non-verbal)	Report span and total correct; cite normative references (e.g., Kessels et al., 2000)	Useful when verbal WM confounded by L2 proficiency; ~5–8 min; low language demands; parallel spatial configurations for repeated testing
Symmetry span	Visuospatial WM capacity; complex span	Computer/tablet; 15–20 min; symmetry judgment + spatial location recall	Partial-credit unit scoring for spatial recall; symmetry accuracy reported separately	Moderate: Requires graphical display capability; validated computerized versions exist	α ≥ 0.70; report both storage and processing scores; cite automated version validation	Alternative to RST when verbal confounds are a concern; ~18–22 min; parallel forms available; administer in sessions without heavy visuospatial AI tasks
Running span	Updating efficiency; WM capacity under continuous input	Computer; 10–15 min; variable-length lists with recall of final n items	Proportion correct at each list length; updating efficiency index	Moderate: Requires variable list programming; less commonly implemented but theoretically important for AI streaming contexts	Report proportion correct by list length; internal consistency; less established norms—pilot validation recommended	Particularly relevant for AI contexts involving continuous information streams (e.g., real-time AI feedback); ~12 min; develop parallel item sets

#### 4.5.4 Integration strategies for AI-mediated research designs

Three primary integration strategies are recommended, depending on research questions, practical constraints, and the temporal granularity required:

1. Pre-Post Module Design. Administer a comprehensive WM battery (e.g., OSPAN + Backward Digit Span + one visuospatial task) at baseline and post-intervention as a separate assessment module. This approach supports aptitude–treatment interaction analyses and enables detection of WM-related change over time. Recommended timing: 35–45 min per assessment occasion; schedule assessments on separate days from intensive AI learning sessions to avoid fatigue confounds.
2. Intermittent Micro-Assessment Design. Embed brief WM tasks (e.g., Backward Digit Span, abbreviated n-back) at strategic points within the AI learning sequence—for example, at the beginning of every third or fourth session. This approach enables tracking of WM fluctuations and state-dependent performance while minimizing learner burden. Recommended timing: 5–10 min per micro-assessment; maintain consistent timing relative to AI task engagement.
3. Triangulated Multi-Method Design. Combine pre-post direct WM assessment with continuous behavioral proxy collection (interaction logs, response latencies, eye-tracking indices) throughout the AI intervention. Analyze convergence and divergence between direct and proxy measures to distinguish task-demand effects from WM-capacity effects. This approach provides the strongest inferential base for understanding whether AI reduces demands, improves utilization, or affects capacity.

#### 4.5.5 Minimum psychometric and reporting standards

To ensure interpretability and cross-study comparability, AI-mediated WM research should adhere to the following minimum reporting standards:

- Internal consistency: Report Cronbach’s *α* or split-half reliability for all WM tasks (minimum acceptable: α ≥ 0.70).
- Validity evidence: Cite validation studies for computerized versions; report correlations with established WM measures if using adapted instruments.
- Language considerations: For verbal WM tasks, specify stimulus language, proficiency requirements for sentence/word stimuli, and any adaptations for L2 populations; pilot with target population to ensure appropriate difficulty.
- Sample descriptives: Report WM score distributions (means, SDs, ranges) to enable meta-analytic integration and comparison with normative data.
- Effect size reporting: Report standardized effect sizes (Cohen’s d, partial η^2^) for WM-related main effects and interactions.

#### 4.5.6 Practical implementation considerations

Timing burden represents a significant practical constraint. Researchers should budget 20–45 min for comprehensive pre-post assessment or 5–10 min for intermittent micro-assessments. Practice effects can be mitigated through parallel forms (available for OSPAN, RST, digit span) or adequate inter-assessment intervals (minimum 2–3 weeks for complex span tasks). For studies involving repeated WM assessment, counterbalancing of parallel forms across participants is essential. Mobile delivery is feasible for most tasks but requires validation of touch-screen response accuracy and careful attention to ambient noise for auditory stimuli. Finally, researcher should consider participant fatigue: avoid scheduling WM assessment immediately after cognitively demanding AI tasks, and monitor for floor or ceiling effects that may indicate inappropriate task difficulty for the sample.

### 4.6 Ethical and risk considerations

The transition to AI-mediated language learning introduces ethical and risk dimensions that extend beyond the equity considerations previously noted and warrant systematic attention from researchers and practitioners. This subsection distinguishes between empirically documented concerns and normative recommendations that, while theoretically grounded, await direct empirical validation in AI-mediated language learning contexts.

#### 4.6.1 Algorithmic bias and fairness risks

Adaptive and generative AI systems inherit potential biases from their training data and algorithmic design, raising fairness concerns for language learners from diverse linguistic, cultural, and socioeconomic backgrounds. Evidence-supported concern: The concentration of AI-mediated research in East Asian EFL contexts (Chinese and Iranian learners) documented in this review indicates that AI systems may be disproportionately optimized for these populations, potentially disadvantaging learners whose L1 backgrounds, learning styles, or cultural contexts differ from those represented in system development and validation samples. Normative recommendation: Researchers should conduct subgroup performance analyses across demographic and linguistic variables (L1 background, proficiency level, gender, socioeconomic status) to detect differential AI effectiveness and report disaggregated outcomes even when overall effects are positive. Until such analyses are routinely conducted, claims about AI’s compensatory benefits for “lower-WM learners” should be interpreted cautiously, as compensation effects may not generalize across all learner subgroups.

#### 4.6.2 Privacy and data governance

AI-mediated language learning systems collect extensive learner data, including interaction logs, response patterns, performance trajectories, and—in biometric-adaptive implementations—physiological and eye-tracking data (Yuan, 2025). Evidence-supported concern: None of the AI-mediated studies in the corpus reported detailed data governance protocols, creating uncertainty about consent procedures, data minimization practices, retention periods, access controls, and policies governing secondary use of learner data. Normative recommendation: Future research should adhere to established data governance principles: (a) informed consent that specifies what data are collected, for what purposes, for how long, and who will have access; (b) data minimization, collecting only data necessary for the stated research or pedagogical purpose; (c) defined retention periods with secure deletion protocols; (d) access controls restricting data to authorized personnel; and (e) explicit policies prohibiting or governing secondary use (e.g., commercial applications, algorithm training beyond the original study). These safeguards are particularly critical when research involves minors or occurs in educational institutions where power asymmetries may compromise voluntary consent.

#### 4.6.3 Transparency and explainability of AI-driven pedagogical decisions

When AI systems make consequential decisions about task difficulty, feedback content, or learning pathways, learners, educators, and researchers have legitimate interests in understanding how and why those decisions are made. Evidence-supported concern: The AI-mediated studies reviewed (biometric-adaptive reading, generative AI writing assistance, AI-orchestrated VR-AR instruction) employed proprietary or opaque algorithms whose decision logic was not fully specified, limiting the ability of researchers to replicate findings or of educators to calibrate pedagogical expectations. Normative recommendation: Research reports should document the decision rules or model architectures governing AI adaptations to the extent permitted by proprietary constraints, explain what learner inputs trigger what system responses, and acknowledge transparency limitations explicitly. For classroom implementation, educators should be provided with interpretable summaries of how the AI system is adjusting instruction and why, enabling informed pedagogical oversight rather than blind reliance on algorithmic recommendations.

#### 4.6.4 Biometric data: heightened sensitivity and safeguards

Biometric-adaptive systems such as those employing eye-tracking and physiological monitoring (Yuan, 2025) collect uniquely sensitive data that can reveal cognitive states, emotional responses, and attentional patterns beyond what learners may intend to disclose. Evidence-supported concern: While Yuan (2025) demonstrated pedagogical benefits of biometric adaptation, the study did not report protocols for biometric data handling, creating uncertainty about whether proportionality (collecting only biometric data necessary for the pedagogical function), opt-out mechanisms (allowing learners to decline biometric monitoring while still accessing instruction), and enhanced security safeguards (encryption, anonymization, restricted access) were implemented. Normative recommendation: Research involving biometric data should apply heightened ethical scrutiny: (a) proportionality assessments demonstrating that biometric collection is necessary and that less intrusive alternatives are insufficient; (b) robust opt-out options that do not penalize learners who decline biometric monitoring; (c) enhanced security protocols including encryption at rest and in transit, anonymization where feasible, and audit trails for data access; and (d) explicit institutional review board (IRB) or ethics committee approval addressing biometric-specific risks. These safeguards are essential for maintaining learner trust and ensuring that the pedagogical benefits of biometric adaptation are not outweighed by privacy intrusions.

#### 4.6.5 Equity and dependency risks

Framing AI as a compensatory tool for learners with lower working-memory capacity invites careful scrutiny of where compensation ends and dependency begins (Silva Barbosa et al., 2023). Evidence-supported concern: If key cognitive operations are consistently offloaded to AI, there is a risk that learners with fewer prior advantages become locked into permanently scaffolded trajectories, while their more advantaged peers learn to leverage AI as an amplifier of already strong executive and metacognitive skills. Normative recommendation: Instructional designs should incorporate planned fading of AI support and explicit metacognitive training to prevent dependency, with longitudinal monitoring of whether AI-supported learners develop autonomous competencies comparable to those achieved through less scaffolded pathways.

#### 4.6.6 Reporting standards for ethical transparency

To enable ethical evaluation and cross-study comparison, AI-mediated language learning research should report the following minimum information: (a) data types collected (interaction logs, performance data, biometric signals, demographic variables); (b) stated purpose for each data type; (c) retention period and deletion protocols; (d) access controls specifying who can access identifiable or sensitive data; (e) subgroup performance checks disaggregated by key demographic and linguistic variables; (f) consent procedures, including provisions for minors and institutional contexts; and (g) IRB/ethics committee approval status with any biometric-specific conditions. These reporting standards are normative recommendations derived from established research ethics principles; their routine adoption would substantially improve the field’s ability to assess the ethical dimensions of AI-mediated instruction.

### 4.7 Limitations

The comparative analysis juxtaposes two bodies of literature that differ not only in technology but also in time, geography, and methodology. The pre-AI corpus spans more than a decade and includes varied populations, settings, and designs; the AI-mediated studies are clustered within a narrow temporal window, geographically concentrated in only two national contexts (China and Iran) and uniformly situated in EFL learning settings (Table 7). Observed differences may therefore reflect broader shifts in educational practice, demographics, or research norms rather than technology alone, and claims about cross-context generalizability should be treated as provisional until tested via explicit moderator and replication designs.

**Table 7:** Geographic distribution, L1 background, educational setting, and AI tool types in the AI-mediated cluster (*k* = 4; total *N* = 1,322).

Dimension	Category	k (studies)	Total N	Notes/examples (from included primary studies)
Geographic context	China	3	1,229	Yu (2025); Yuan (2025); Fan and Yao (2025)
	Iran	1	93	Esfandiari and Allaf-Akbary (2024)
Learner L1 background	Chinese L1 (variety not reported; inferred from study context)	3	1,229	All three China-based EFL samples
	Iran-based EFL sample (L1 not explicitly reported; context implies Persian/Farsi)	1	93	Iranian advanced EFL sample
Educational setting	EFL (all studies)	4	1,322	No primary AI-mediated studies in ESL settings in this corpus
	University/tertiary explicitly reported	1	546	Fan and Yao (2025)
	Institutional EFL; level not clearly specified	3	776	Yu (2025); Yuan (2025); Esfandiari and Allaf-Akbary (2024)
AI tool types *(non-mutually exclusive)*	Generative LLM/chatbot-mediated learning	3	1,022	ChatGPT-4; DeepSeek V3.1; Microsoft Copilot (GPT-4-based)
	Biometric-adaptive reading platform	1	300	Smart Sparrow + eye-tracking/physiological indicators
	VR–AR multimodal instruction with AI orchestration	1	383	Unity/Oculus/MediaPipe + ChatGPT-4
	AI-mediated intelligent DDL	1	93	Copilot-based DDL vs. AntConc-based DDL

Categories are coded from the four included primary AI-mediated studies in this review; in several cases, L1 and educational level are not explicitly reported and are therefore indicated as inferred/unspecified to make reporting gaps transparent. Tool-type categories can overlap within the same study.

Table 7 clarifies that the AI-mediated evidence base in the present corpus is geographically and culturally narrow, with the large majority of primary evidence drawn from Chinese EFL learners and the remainder from an Iranian EFL sample. This concentration matters because AI–WM effects are plausibly moderated by (a) educational culture and assessment norms (e.g., exam orientation, academic-integrity policy, and the evaluative stakes attached to AI-assisted writing), (b) differential technology access and institutional constraints on AI availability (device access, connectivity reliability, platform restrictions), (c) L1 typology and orthographic depth (which shape baseline decoding/encoding demands and therefore the relative burden on phonological-loop vs. visuospatial resources), and (d) classroom ecology (teacher mediation, class size, peer-collaboration norms, and the extent to which AI use is coached vs. left implicit). Accordingly, the present synthesis should be interpreted as strongest for comparable EFL contexts and should not assume transportability to ESL environments or underrepresented linguistic communities without explicit tests of these moderators.

### 4.8 Future directions

Several lines of inquiry emerge as priorities for advancing understanding of working memory in AI-mediated language learning; however, progress now depends less on additional conceptual claims and more on a reproducible methodological program that can adjudicate among competing mechanisms (task-demand reduction, functional WM utilization, and durable WM change). Accordingly, future work should replace general calls with a methodological blueprint specifying (a) recommended designs, (b) minimum measurement sets, (c) analysis expectations, and (d) reporting standards that collectively enable cumulative, comparable evidence across AI tools, skills, and learning ecologies.

Recommended designs. Future studies should prioritize randomized controlled trials (RCTs) that incorporate baseline WM stratification and aptitude–treatment interaction (ATI) logic. At minimum, trials should (i) administer validated WM measures at baseline and use blocked/stratified randomization to balance WM distributions across conditions (or prespecify WM × condition moderation as the primary ATI test), (ii) include an active comparator rather than “no-treatment” controls (e.g., non-AI digital support or teacher-guided support matched for time-on-task), and (iii) implement longitudinal measurement with pretest, immediate posttest, and delayed follow-up (e.g., 4–8 weeks) to distinguish short-term performance effects from retention and transfer. Where interventions are delivered in intact classes or schools, cluster RCTs or classroom-embedded individual randomization should be used with analytic correction for nesting. Where the research question concerns immediate interface-level regulation (e.g., adaptive vs. non-adaptive reading), crossover/within-subject designs are appropriate, provided that order effects and carryover are mitigated through counterbalancing and washout periods.

Minimum measurement set. To close the measurement paradox identified in the AI literature, AI-mediated studies should adopt a minimum measurement bundle that includes (1) direct WM tasks, (2) language outcomes aligned to the targeted skill, and (3) process data that captures how learners allocate effort in human–AI interaction. For WM, studies should include at least one validated verbal complex-span index and one brief manipulation/updating index (with parallel forms where feasible), administered at baseline and, when claims involve WM change, repeated post-intervention and at delayed follow-up (see Table 6 for validated computerized options). For outcomes, studies should include skill-appropriate performance measures (e.g., writing quality/accuracy/complexity indices for AI-assisted writing; comprehension and inference measures for adaptive reading; vocabulary form–meaning mapping and delayed retention for VR–AR multimodal instruction), with immediate and delayed tests to quantify retention. For process data, studies should minimally capture interaction traces (e.g., prompt sequences, revision histories/keystroke logs, time-on-task, response latencies), supplemented where feasible by validated cognitive-load instruments and/or physiological proxies (e.g., eye-tracking indices in adaptive reading) to distinguish load reduction from load redistribution.

Analysis expectations. Analyses should be planned explicitly to test ATI/moderation and ecological nesting. WM should be treated as a continuous moderator when possible (to avoid information loss), with prespecified WM × condition interaction terms as primary tests of instructional fit. In classroom/ecological implementations, multilevel models should be used to account for nesting (learners within classes/teachers/schools) and repeated measures (pre/post/follow-up). Where AI is hypothesized to operate via load redistribution, mediation models should be prespecified to test whether process indicators (e.g., prompt-management burden, revision depth, fixation/regression patterns) explain AI effects on language outcomes, while separating these pathways from baseline proficiency and motivation. Missing data and attrition should be handled transparently (e.g., mixed-effects models with maximum likelihood, sensitivity analyses, and/or multiple imputation), and primary analyses should follow intention-to-treat principles to avoid inflation of AI effects through selective compliance.

Reporting standards for reproducibility and risk-of-bias control. Reports should document randomization procedures (unit of randomization, allocation method, and concealment where applicable), comparator conditions (content, time-on-task, and instructor involvement), attrition and adherence (with reasons and condition-wise flow), and prespecified outcomes/moderators (with preregistration when feasible). Because AI systems change rapidly, intervention fidelity must include AI-specific transparency: the tool name, model/version (or release date), access mode (web/app/API), prompting constraints (templates, guardrails), and the exact scaffolds given to learners (e.g., evaluation checklists, prompt training). When human ratings are used (e.g., writing quality), rater blinding to condition and reliability indices should be reported. Finally, risk-of-bias safeguards should be explicit (baseline equivalence checks, contamination controls between conditions, and protocol deviations), enabling credible cross-study synthesis.

Exemplar protocol 1 (ATI-stratified classroom RCT: generative AI-assisted writing). A next-step study could recruit multiple intact EFL classes across at least two institutions to ensure ecological validity. Learners would complete a baseline WM battery (verbal complex span plus a brief manipulation/updating task) and a baseline writing assessment. Within each class, learners would be stratified by baseline WM (e.g., tertiles or continuous blocking) and then randomized to (a) generative AI-assisted writing with an explicit metacognitive evaluation routine (prompt-planning + output-verification checklist) or (b) a matched non-AI digital writing support condition (e.g., teacher-provided exemplars and feedback cycles matched for time-on-task). Instruction would run for 6–8 weeks with standardized task prompts and equivalent writing opportunities. Outcomes would include immediate posttest writing performance and a delayed follow-up writing task, while process data would include prompt sequences, revision histories/keystroke logs, and a validated cognitive-load profile during writing. The primary analysis would be a multilevel longitudinal model (learners nested within classes) testing time × condition effects and the prespecified WM × condition ATI interaction; secondary analyses would test whether process measures (e.g., revision depth, prompt-management burden) mediate condition effects on writing outcomes, clarifying whether AI benefits operate through demand reduction or load redistribution.

Exemplar protocol 2 (randomized crossover: biometric-adaptive vs. non-adaptive AI reading). To isolate preemptive regulation effects while controlling stable individual differences, a crossover design could assign learners to two counterbalanced sequences: (A) biometric-adaptive AI reading for 2–3 weeks followed by non-adaptive AI reading for 2–3 weeks, or (B) the reverse order, with a short washout period and matched text difficulty bands. Baseline WM would be measured prior to the first phase, and reading comprehension plus delayed retention would be assessed at the end of each phase. Process measures would include eye-tracking/reading-time indicators and validated cognitive-load subscales captured repeatedly during reading sessions. Analysis would use mixed-effects models including condition, period, and order effects, with WM as a prespecified moderator to test whether preemptive adaptation disproportionately benefits lower-WM learners (a compensatory hypothesis) or instead shifts burden toward executive monitoring uniformly. This design would directly operationalize the causal question that current AI studies leave unresolved: whether improved performance reflects reduced task demands, improved WM utilization, or WM-linked differential responsiveness to adaptive regulation.

## 5 Conclusion

This review has traced the evolution of working memory as a central construct in technology-enhanced language learning across two technological epochs. In the pre-AI era, working memory functioned as a hard constraint: an internal bottleneck around which well-designed systems attempted to navigate. In the emerging AI-mediated era, it becomes part of a distributed, dynamically regulated system in which cognitive demands can be sensed, reallocated, and, potentially, reshaped through continuous interaction with intelligent tools.

The analysis suggests that AI’s most consequential contribution is not simply its capacity to deliver content more efficiently, but its ability to reorganize the division of cognitive labor between human and machine. By offloading some processes and intensifying others, AI forces a reconceptualization of what it means to design, teach, and learn in ways that are sensitive to working-memory limits and possibilities. At the same time, the methodological apparatus of the field has not yet caught up with these conceptual advances. Without systematic, direct measurement of working memory and rigorous tests of how it interacts with AI affordances, claims about compensation, optimization, and training will remain aspirational.

The challenge, then, is twofold. Conceptually, the field must embrace a view of learning in which working memory is neither a fixed liability to be accommodated nor a simple trait to be correlated, but a dynamic resource that co-evolves with the tools through which learners engage with language. Methodologically, it must commit to an empirical program capable of illuminating the hidden dynamics of this human–AI collaboration. Only by holding these two commitments together can AI-mediated language learning move from speculative promise to evidence-based practice—and, in doing so, transform working memory from a limiting boundary into a central site of educational design and innovation.

## Data Availability

The original contributions presented in the study are included in the article/Supplementary material, further inquiries can be directed to the corresponding author.
