# Supplementary material for: Working memory in technology-enhanced language learning: a systematic review from interactive to AI-mediated contexts
Source: Front Psychol. 2026 Feb 18;17:1758104. doi: 10.3389/fpsyg.2026.1758104 (PMC12957077; doi:10.3389/fpsyg.2026.1758104)
Supplement: Supplementary file 1 [file Supplementary_file_1.docx]

**Appendix A**

**PRISMA 2020 Checklist and Location of Information in the Manuscript**

**Table A1**

*PRISMA 2020 checklist with location of information in the manuscript*

| **Section/Topic** | **Item** | **Checklist item (PRISMA 2020; paraphrased)** | **Location in manuscript** |
| --- | --- | --- | --- |
| Title | 1 | Identify the report as a systematic review. | Title page (p. 1). |
| Abstract | 2 | Provide a structured abstract covering objectives, methods, results, and conclusions. | Abstract (p. 2). |
| Introduction | 3 | Explain the rationale for the review in the context of existing knowledge. | Introduction (pp. 3-6). |
| Introduction | 4 | State the review objectives/questions explicitly. | Introduction (p. 6; paragraph beginning “Against this backdrop…”). |
| Methods | 5 | Specify inclusion and exclusion criteria. | Method: Phase 1 eligibility criteria (p. 8) and Phase 2 eligibility criteria (pp. 9-10). |
| Methods | 6 | Specify all information sources (databases and other methods) and the date range searched. | Method: Phase 1 sources and date limits (p. 7); Phase 2 sources and search window through 2025 (pp. 8-9). |
| Methods | 7 | Present full search strategies for each database/source (replicable). | Method: Phase 1 core search string and database adaptation (p. 7); Phase 2 AI-specific strings and targeted searches (pp. 8-9). |
| Methods | 8 | Describe the study selection process (screening/eligibility decisions; how disagreements were resolved). | Method: screening and eligibility workflow with counts (p. 8); resolution procedure noted for quality appraisal (p. 12). |
| Methods | 9 | Describe the data collection/extraction process (who extracted; how verified). | Method: standardized data-extraction template and extracted fields (p. 11). |
| Methods | 10a | List and define outcomes sought. | Method: outcome measures recorded (p. 11). |
| Methods | 10b | List and define other variables and data items collected (participants, setting, technology, WM constructs/measures). | Method: participant, context, technology, WM constructs/measures, design, and key findings fields (p. 11). |
| Methods | 11 | Describe methods used to assess risk of bias/quality of included studies. | Method: MMAT, Cochrane RoB, and ROBINS-I procedures (p. 12). |
| Methods | 12 | Specify effect measures used for synthesis (or state not applicable if no meta-analysis). | Not applicable (no pooled effect estimate/meta-analysis); historical-comparative synthesis stated in Method (p. 7) and analysis approach (pp. 11-12). |
| Methods | 13a | Describe how studies were grouped and compared for synthesis. | Method: within-era synthesis and historical-comparative integration (pp. 11-12). |
| Methods | 13b | Describe any data preparation steps (e.g., conversions, handling missing summary data). | Not reported as a distinct step; extraction fields and statistical-detail requirements described (p. 10-11). |
| Methods | 13c | Describe methods used to display results (tables/figures) and summarize evidence. | Method: corpus structure summary (Table 1; p. 12) and Results narrative structure (pp. 13-20). |
| Methods | 13d | Describe methods used to synthesize results (narrative/thematic/comparative framework). | Method: two-stage synthesis (within-era themes; cross-era comparative dimensions) (pp. 11-12). |
| Methods | 13e | Describe methods to explore heterogeneity (if applicable) or justify qualitative comparison approach. | Not applicable for statistical heterogeneity (no meta-analysis); comparative dimensions specified in Method (pp. 11-12). |
| Methods | 13f | Describe sensitivity analyses (if performed) or state none. | Not performed (no sensitivity analyses reported). |
| Methods | 14 | Describe any methods used to assess reporting bias or publication bias. | No formal assessment conducted; publication bias noted from contextual meta-analysis evidence (Method, p. 12; Limitations, p. 24). |
| Methods | 15 | Describe methods used to assess certainty/strength of evidence (e.g., GRADE) or state not assessed. | Not assessed (no certainty framework applied; not reported as performed). |
| Results | 16a | Report results of the search and selection process (counts at each stage). | Method: selection counts (p. 8). PRISMA flow diagram should be reported as Figure 1 (insert in Method section; after Phase 2 inclusion statement). |
| Results | 16b | Cite and report full-text exclusions with reasons (ideally with counts). | Full-text exclusion count reported in Method (p. 8). Categorized reasons with counts should be reported in Figure 1 (PRISMA flow diagram). |
| Results | 17 | Present characteristics of included studies. | Results narrative describing study designs, contexts, and interventions (pp. 13-20) and corpus summary (Table 1; p. 12). |
| Results | 18 | Present risk-of-bias/quality assessments for included studies. | Method: tools and appraisal process reported (p. 12). (Recommended: add a study-level quality summary table; cite location here once inserted.) |
| Results | 19 | Present results of individual included studies (as appropriate). | Results (pp. 13-20). |
| Results | 20a | Summarize characteristics and risk of bias of studies contributing to each synthesis. | Results synthesis by corpus/era (pp. 13-20); limitations and quality considerations noted (Method, p. 12; Limitations, pp. 24-25). |
| Results | 20b | Present results of any statistical syntheses (meta-analysis) and heterogeneity metrics, if conducted. | Not applicable (no meta-analysis; narrative/historical-comparative synthesis). |
| Results | 20c | Present results of investigations of heterogeneity (subgroup/meta-regression), if conducted. | Not applicable (no heterogeneity analyses reported). |
| Results | 20d | Present results of sensitivity analyses, if conducted. | Not applicable (no sensitivity analyses reported). |
| Results | 21 | Present results of reporting bias assessment, if conducted. | No formal assessment conducted; publication bias risk discussed (Method, p. 12; Limitations, p. 24). |
| Results | 22 | Present certainty/strength of evidence for outcomes, if assessed. | Not assessed (no certainty framework applied). |
| Discussion | 23a | Interpret findings in the context of other evidence. | Discussion (pp. 21-22). |
| Discussion | 23b | Discuss limitations of the evidence base. | Limitations (pp. 24-25). |
| Discussion | 23c | Discuss limitations of the review process. | Limitations (pp. 24-25). |
| Discussion | 23d | Discuss implications for practice, policy, and research. | Implications (p. 23) and Future Directions (pp. 25-26). |
| Other information | 24a | Provide registration information (register name and number) or state not registered. | Appendix A: Declarations (after Table A1). |
| Other information | 24b | Indicate where the protocol can be accessed, or state no protocol was prepared/published. | Appendix A: Declarations (after Table A1). |
| Other information | 24c | Describe and explain any amendments to the protocol (or state none). | Appendix A: Declarations (after Table A1). |
| Other information | 25 | Describe sources of support and the role of funders. | Appendix A: Declarations (after Table A1). |
| Other information | 26 | Declare competing interests. | Appendix A: Declarations (after Table A1). |
| Other information | 27 | Report availability of data, code, and other materials (e.g., extraction sheet, search strings). | Appendix A: Declarations (after Table A1). |

Note. WM = working memory; CALL = computer-assisted language learning; VR-AR = virtual reality and augmented reality; LLM = large language model; DDL = data-driven learning; ATI = aptitude-treatment interaction.

**Declarations (PRISMA 2020 Items 24-27)**

Registration and protocol. The review protocol was not registered, and no publicly accessible protocol is available. The two-phase historical-comparative design required iterative refinement of Phase 2 eligibility criteria as the AI-mediated literature emerged during 2024-2025.

Support. The authors report that no external funding was received for this work.

Competing interests. The authors declare that they have no competing interests.

Availability of data, code, and other materials. The database search strings and data-extraction template are available in the Supplementary Materials. The extracted dataset is available from the corresponding author upon reasonable request.
